# A nonlinear exaggerated kernel framework for expressive hair motion in tracking solution-based simulation

**DOI:** 10.1371/journal.pone.0356631

**Published:** 2026-08-31

**Authors:** Dohee Park, Jong-Hyun Kim

**Affiliations:** 1 College of Software and Convergence (Dept. of Design Technology), Inha University, Michuhol-gu, Incheon, South Korea; 2 College of Software and Convergence (Dept. of Artificial Intelligence, Design Technology), Graduate School of Electrical and Computer Engineering, Inha University, Michuhol-gu, Incheon, South Korea; PLOS, UNITED KINGDOM OF GREAT BRITAIN AND NORTHERN IRELAND

## Abstract

This paper proposes a nonlinear exaggerated kernel framework for controlling the motion of ghost strands in a more expressive and stable manner in tracking solution-based hair simulation. Conventional kernel-based interpolation methods restrict interpolation coefficients between 0 and 1, which makes it difficult to sufficiently represent nonlinear hair motions such as swaying, overlapping, and splitting of ghost strands, even when large momentum is applied to guide strands. In particular, for ghost strands located between two target strands, a problem arises where motion is excessively reduced due to momentum cancellation. To address this issue, this study introduces the concept of high-frequency component enhancement from signal processing and designs an exaggerated kernel that combines a sigmoid-based low-frequency response with a nonlinear high-frequency response. The proposed kernel allows interpolation coefficients to exceed 1, thereby nonlinearly amplifying the motion of ghost strands in regions where the relative motion direction difference between strands is large. In addition, to alleviate the strand length increase problem that may occur due to exaggerated interpolation, an absolute-value-based exaggerated kernel and a positive-component-constrained kernel are introduced to ensure physical stability. Furthermore, considering that the root and tip of hair have different dynamic characteristics, a root–tip weighted kernel framework with different frequency characteristics depending on position is proposed. Through this, stable motion is maintained near the root, while natural hair motion with emphasized swaying and splitting can be effectively expressed near the tip. Through various experimental results, it is confirmed that the proposed method is superior in terms of visual expressiveness and dynamic diversity compared to conventional kernel-based tracking solutions, and it is shown that the expressiveness of hair motion can be effectively improved without modifying the physical simulation model.

## Introduction

Realistic hair simulation is one of the key factors that determine visual quality across character animation, virtual humans, games, films, and immersive content. However, physically simulating tens of thousands to hundreds of thousands of hair strands poses significant computational constraints. For this reason, tracking solutions have been widely used, in which only a small number of guide strands are simulated using physics-based methods, and the remaining large number of detail strands determine their motion by following them.

In tracking solution-based hair simulation, the interpolation process that defines the relationship between guide strands and detail strands greatly affects the quality of the visual result. In particular, when a single ghost strand tracks two or more target strands, the expressiveness of nonlinear hair motions such as swaying, overlapping, and splitting varies significantly depending on how interpolation weights are designed. The simplest step function-based interpolation can clearly separate motion between strands, but lacks continuity and produces unnatural motion. On the other hand, continuous kernels such as the sigmoid function provide smooth interpolation, but since the interpolation weights are restricted between 0 and 1, hair motion becomes excessively damped even when large momentum is applied, resulting in reduced expressiveness.

This limitation is particularly evident for ghost strands located between two target strands. When the motion directions of the two target strands are opposite, conventional kernel-based interpolation causes momentum cancellation, leading to abnormally weakened motion of ghost strands, and consequently, swaying and splitting appear only weakly. This is not a problem of the physical simulation itself, but originates from the kernel design in which the distribution of interpolation weights is flattened in a low-frequency manner in the mid-region. Therefore, reconsideration of the kernel function itself is required.

In this paper, to address this problem, we introduce the concept of high-frequency enhancement widely used in signal processing into the design of hair strand interpolation kernels (here, high-frequency components refer not to temporal oscillation frequencies, but to regions with large spatial variation in interpolation weights). While conventional sigmoid kernels provide smooth low-frequency variation, the proposed nonlinear exaggerated kernel selectively amplifies regions where the change in interpolation weights is large, i.e., high-frequency regions, thereby nonlinearly emphasizing the relative motion difference between target strands. Through this, even while allowing interpolation weights to exceed 1, natural overlapping and splitting of hair strands can be effectively represented.

However, exaggeration of interpolation weights may simultaneously cause excessive increase in strand length or physical instability. To address this, this study analyzes the velocity cancellation and amplification behavior that may occur when applying the exaggerated kernel, and proposes methods to maintain physical stability using an absolute-value-based exaggerated kernel and a positive-component-constrained kernel. In addition, considering that the root and tip of hair have different dynamic characteristics, we propose a root–tip weighted kernel framework that applies kernels with different frequency characteristics depending on position.

The main contributions of this paper can be summarized as follows. First, we analyze the structural limitations of conventional kernel interpolation that restrict the expressiveness of ghost strands in tracking solution-based hair simulation, and propose a nonlinear exaggerated kernel to address this issue. Second, by systematically applying the concept of high-frequency enhancement to kernel design, we effectively amplify swaying and splitting of hair motion without modifying the physical simulation model. Third, we propose stabilization techniques and a root–tip weighted framework to control physical instability caused by exaggerated interpolation, and validate their effectiveness through various experiments. Based on this background, this study aims to systematically analyze the structural impact of interpolation kernels on the motion expressiveness of ghost strands in tracking solution-based hair simulation.

### Problem statement

In tracking solution-based hair simulation, a small number of target (guide) strands determine motion through physical simulation, and a large number of ghost strands compute their motion by interpolating the physical quantities of these target strands. In this process, the interpolation coefficient applied to each ghost strand is a key factor that determines how much of the target strand motion is reflected, and the expressiveness of the resulting hair motion strongly depends on the spatial distribution of these interpolation coefficients.

In general, kernel-based interpolation is designed so that the interpolation coefficient *t* lies within the range [0,1]. This constraint is advantageous for ensuring numerical stability and physical intuition, but introduces structural limitations when tracking two or more target strands simultaneously. In particular, when the motion directions of two target strands are opposite, the interpolated velocity vector of ghost strands in the mid-region is canceled out, resulting in significantly smaller motion compared to the actual input momentum.

Formally, let the velocities of two target strands *A* and *B* be ${𝐯}_{A}$, ${𝐯}_{B}$, respectively. The interpolated velocity of a ghost strand is computed as:


$$\begin{array}{c} {𝐯}_{interp}={𝐯}_{A}\cdot t+{𝐯}_{B}\cdot (1-t), \end{array}$$ (1)

When ${𝐯}_{A}=-{𝐯}_{B}$, if t≈0.5 in the mid-region, then ${𝐯}_{interp}\approx 0$, and the momentum is almost completely canceled.

**Fig 1:**
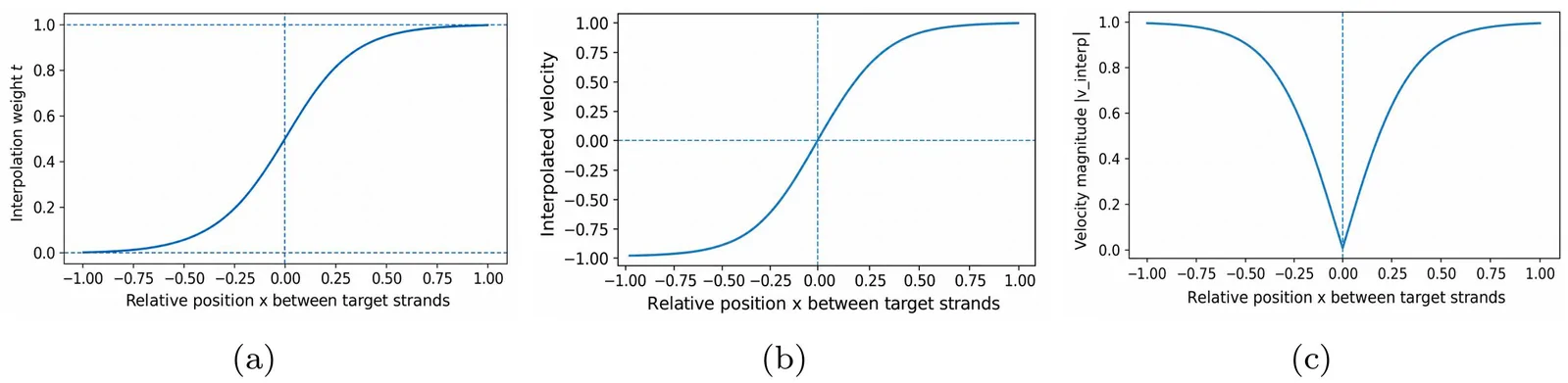
Structural limitation of conventional kernel-based interpolation. (a) Sigmoid interpolation weight constrained to t∈[0,1]. (b) Interpolated velocity when two target strands move in opposite directions, showing velocity cancellation near the middle region. (c) Magnitude of the interpolated velocity, where the motion of ghost strands becomes weakest between the targets.

Fig 1a shows the typical distribution of the interpolation coefficient *t*(*x*) using a sigmoid kernel. The interpolation coefficient is always constrained within [0,1], and takes a value of t≈0.5 at the midpoint between two target strands. Fig 1b shows the interpolated velocity when this interpolation coefficient is applied to two target strands moving in opposite directions, and the cancellation phenomenon where the velocity converges to zero in the mid-region is clearly observed. Fig 1c visualizes the magnitude of velocity $\left|{𝐯}_{interp}\right|$, showing that the motion of ghost strands becomes weakest in the mid-region between the target strands, revealing a structural problem.

This phenomenon is not simply a problem of parameter tuning, but originates from the kernel design itself, where the interpolation coefficient is restricted within [0,1]. In the mid-region, the variation of interpolation coefficients is small, and the relative motion difference between target strands is flattened in a low-frequency manner, so that high-frequency nonlinear motions such as swaying, overlapping, and splitting, which are important in hair simulation, are effectively suppressed. As a result, ghost strands located inside hair clusters repeatedly exhibit abnormally weakened motion.

Therefore, to fundamentally improve the expressiveness of ghost strands in tracking solution-based hair simulation, instead of adjusting only the slope of the kernel while fixing interpolation coefficients within [0,1], a new kernel framework is required that can redesign the distribution and variation of interpolation coefficients so as to alleviate momentum cancellation in the mid-mixing region.

### Effect of exaggerated kernel on mid-region cancellation

**Fig 2:**
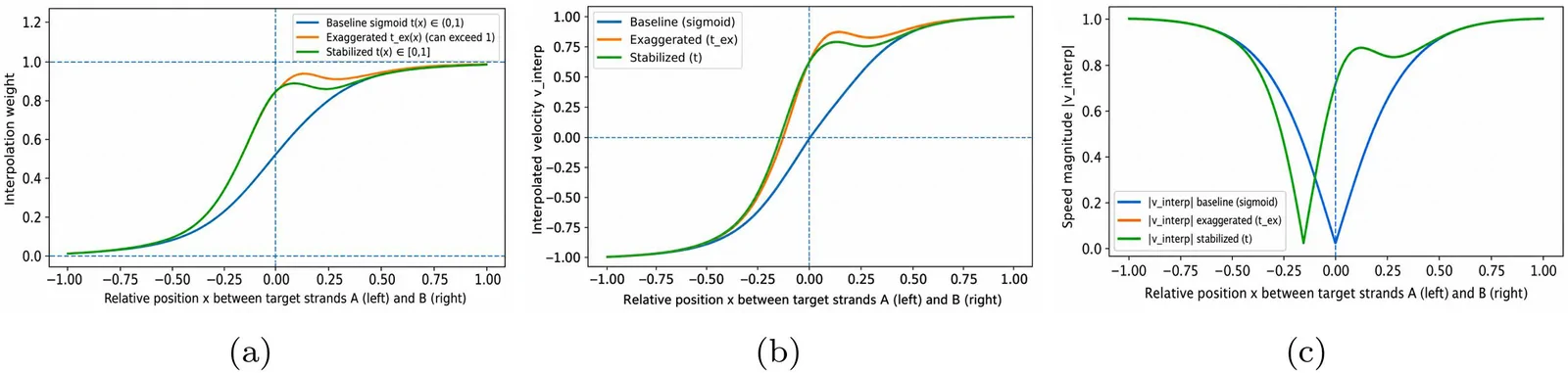
Kernel comparison and cancellation analysis. (a) Kernel comparison among the baseline sigmoid kernel *t*(*x*), the exaggerated kernel $t_{ex}(x)$, and the stabilized kernel $\tilde{t}(x)$. Note that $\tilde{t}(x)$ closely overlaps with $t_{ex}(x)$ over most regions and may appear visually indistinguishable in the plot, indicating that stabilization preserves the exaggeration effect while enforcing bounded weights. (b) Interpolated velocity under opposite target motions ($v_{A}=+1$, $v_{B}=-1$), where the baseline exhibits mid-region cancellation while the exaggerated/stabilized kernels avoid the collapse. (c) Speed magnitude $\left|v_{interp}\right|$ showing reduced mid-region cancellation with the exaggerated kernel design.

Fig 2 visually shows the mid-region momentum cancellation problem that occurs in conventional kernel-based interpolation when two target strands move in opposite directions, and the effect of the exaggerated kernel design to alleviate this problem.

Fig 2a compares the shapes of three interpolation kernels. The conventional sigmoid kernel *t*(*x*) is strictly limited to the range t(x)∈(0,1) and provides smooth and stable interpolation, but enforces t(x)≈0.5 in the mixing region between two target strands. As a result, ghost strands located in the mid-region track the motion of the two target strands by mixing them in nearly equal proportions.

In contrast, the exaggerated kernel $t_{ex}(x)$ is designed to allow $t_{ex}(x)>1$ in a limited region by adding localized high-frequency components to the mid-mixing region. This design intentionally shifts the interpolation coefficient away from 0.5 in the mid-region, thereby structurally alleviating the momentum cancellation that occurs in conventional kernels. In addition, the stabilized kernel $\tilde{t}(x)$ applies absolute-value-based weighting and normalization, maintaining the range $\tilde{t}(x)\in [0,1]$, while being designed to have a sharper transition than the conventional sigmoid kernel.

Fig 2b compares the interpolated velocity $v_{interp}$ when the velocities of the two target strands are opposite, i.e., $v_{A}=+1$, $v_{B}=-1$. When using the conventional sigmoid kernel, t(x)≈0.5 in the mid-region, so the interpolated velocity converges to $v_{interp}\approx 0$, and the motion of the ghost strand effectively disappears. In contrast, when applying the exaggerated and stabilized kernels, the interpolated velocity does not pass through zero in the same region, and maintains a meaningful value. This indicates that simply redesigning the distribution of interpolation coefficients can effectively suppress the cancellation phenomenon in the mid-region.

Fig 2c shows the magnitude of the interpolated velocity $\left|v_{interp}\right|$. In the conventional sigmoid kernel, the velocity magnitude sharply decreases in the mid-region, leading to a structural problem where the motion of ghost strands becomes weakest. In contrast, the exaggerated kernel maintains a relatively high value of $\left|v_{interp}\right|$ even in the same region, so that splitting and fluttering of ghost strands appear more clearly. The stabilized kernel shows a similar tendency, demonstrating that mid-region cancellation can be alleviated while maintaining numerical stability.

These results suggest that the loss of expressiveness observed in conventional kernel-based tracking solutions is not simply a matter of parameter tuning, but arises from the structural limitation of kernel design that restricts interpolation coefficients to the range [0,1]. Therefore, emphasizing high-frequency components in the mid-mixing region and redesigning the distribution of interpolation coefficients through an exaggerated kernel can be considered a key approach for inducing richer and more expressive strand motion in tracking solution-based hair simulation. The detailed contributions are summarized below.

### Contributions

This section briefly summarizes the contributions of this study. First, we analyze that kernel interpolation constrained to t∈[0,1] structurally causes mid-region cancellation under opposite directional motion, and formalize this phenomenon from the perspective of kernel design. In addition, we combine a nonlinear exaggerated kernel that selectively amplifies high-frequency transition regions with stabilization techniques that suppress negative weights and excessive amplification, thereby achieving both expressiveness and numerical stability. Furthermore, through a root–tip adaptive exaggeration strategy, we enable control that reflects dynamic characteristics depending on strand position. Finally, the proposed method is designed as a lightweight extension layer that is applied only to the guide–detail mapping stage without modifying the existing physical solver, making it easy to integrate into real-time production pipelines.

## Related work

This section reviews related work focusing on guide–detail structures, strand interpolation and control, and kernel/constraint-based simulation frameworks in tracking solution-based hair simulation.

### Strand- and continuum-based hair dynamics models

Hair simulation can be broadly categorized into approaches that directly model individual strands and those that approximate hair as a continuum. From the continuum perspective, hair volume is treated as a fluid or continuous medium to model interactions and alleviate large-scale contact problems [6,7]. On the other hand, at the individual strand level, large-scale hair can be directly simulated using mass–spring systems [8], or wisp-based frameworks have been proposed to robustly handle contact and collisions [9]. In addition, modeling strands as elastic rods provides more accurate mechanics including twisting and bending, and Discrete Elastic Rods, in particular, present a stable simulation framework through geometric discretization of thin, elongated rods [10]. This rod-based perspective has also been extended to Super-Helices models for predicting natural hair dynamics [11,12]. For real-time applications, methods have been proposed to accelerate rod/spring models using GPUs or to incorporate constraints for style preservation [13,14].

### Hair structuring, clustering, and wisp control

Since hair exhibits strong clustering behavior, many approaches aim to control macroscopic motion using clusters or wisps. Adaptive Wisp Tree, which models splitting and merging of hair clusters using a multiscale structure, provides a classical foundation for dynamic clustering control [15]. In addition, methods have been proposed to approximate hair as particle sets for fast animation in interactive environments [16], and frameworks have been developed to robustly handle collisions while managing complex hairstyles at the wisp level [9]. From a modeling perspective, multiresolution editing methods have been proposed to construct hierarchical cluster structures [17], which have influenced subsequent guide-based control and LOD design.

### Guide–detail structures and tracking solutions

To address the computational cost of large-scale hair, guide–detail structures are widely used, where only a small number of guide strands are simulated, and the remaining detail strands are interpolated or tracked. In this context, HairControl constructs constraints so that high-resolution hair follows input motion (guides) in a spatially averaged sense, presenting a tracking solution that combines artistic intent with physically-based detail [1]. In addition, reduced model approaches have been proposed, which approximate hair motion in a low-dimensional space based on precomputed data, simulating only a small number of guides at runtime while interpolating the rest [2]. Recently, guide-based structures have been extended to neural interpolation methods to achieve real-time quality across various hairstyles [3]. Meanwhile, methods have also been proposed to construct simulation-ready hair by estimating material properties from dynamic capture data [18].

### Constraint-based simulation and numerical stability

Hair is a thin and elongated structure, so constraints such as inextensibility, bending, and twisting are important, and numerical stability is a key issue in real-time applications. Position-Based Dynamics (PBD) has been widely adopted for real-time simulation due to its stability and controllability based on position projection [4], and methods have been proposed to efficiently simulate quasi-inextensible hair and fur [19]. In addition, XPBD has been introduced to alleviate stiffness and time-step dependency issues in PBD, enabling more consistent constraint handling based on compliance [5]. Projective Dynamics combines constraint projection with an energy minimization framework, providing an efficient implicit integration scheme [20], and is highly applicable to strongly constrained structures such as hair. For real-time hair simulation, methods have also been proposed to model interactions within volume using hair meshes or sheet representations instead of individual strands [21]. Furthermore, inverse approaches have been proposed to estimate forces and contacts that explain target motion by solving hair dynamics with frictional contact [22].

Table 1 compares related work in tracking solution-based hair simulation based on guide–detail mapping (interpolation/tracking) strategies. The simplest hard assignment (e.g., nearest guide selection) is computationally efficient and suitable for real-time applications, but suffers from discontinuities due to abrupt guide switching, making it difficult to represent nonlinear motions such as splitting and fluttering. In contrast, continuous kernel-based interpolation such as sigmoid functions provides visually smooth transitions, but due to the constraint t∈[0,1], the mid-mixing region is forced to t≈0.5, leading to a structural “dead zone” where interpolation velocities cancel out when guides move in opposite directions.

**Table 1:** Comparison of guide-to-detail mapping strategies for tracking-solution-based hair simulation. Symbols ✓/△/× indicate strong/moderate/weak performance with respect to each criterion.

Method Category	Representative Work	Blending / Control Cue	Expressive Splitting	Mid-Region Stability	Real-Time Suitability	Key Limitations
Hard assignment (nearest guide)	Guide-following in production pipelines (generic)	Nearest-guide / step-like selection	×	△	✓	Abrupt switching and visual discontinuity; weak fluttering and minor splitting
Bounded smooth kernel interpolation	Sigmoid-style bounded blending (generic)	Smooth kernel with t∈[0,1]	△	×	✓	Velocity cancellation in the mixing region when guides move oppositely (“dead zone”)
Tracking solution with constraints	HairControl [1]	Constraint-based tracking / space-averaged goals	△	△	△	Requires careful constraint design; may still under-emphasize nonlinear splitting under opposing motions
Reduced/Model-order approaches	Reduced interactive hair [2]	Low-dimensional basis / reduced coordinates	△	△	✓	Quality depends on training/basis coverage; limited extrapolation under extreme motions
Learning-based interpolation	Neural interpolation [3]	Learned latent features for guide-to-detail mapping	✓	△	✓	Data dependence; limited interpretability and controllability of specific mid-region cancellation
Constraint-based real-time dynamics (backbone)	PBD/XPBD [4,5]	Position-level constraints / compliant constraints	–	✓	✓	Not an interpolation method by itself; still needs a robust guide-to-detail mapping
**Ours**	**This work**	**High-frequency emphasized exaggerated kernel, stabilization, and root–tip adaptation**	✓	✓	✓	Mitigates mid-region cancellation by reshaping kernel transition while maintaining stability

Tracking solution approaches such as HairControl provide directional control by tracking detail strands through constraint-based objectives, but have limitations in directly alleviating mid-region cancellation caused by opposite guide motions from the perspective of kernel design. Reduced model approaches can achieve real-time performance with fewer degrees of freedom, but their expressiveness in extreme motions may be limited depending on the coverage of precomputed bases. Learning-based interpolation can provide high-quality details, but suffers from data dependency and lacks interpretability and controllability for specific phenomena such as mid-region cancellation.

In contrast, this study does not modify the physical simulation backbone (PBD/XPBD, etc.), but restructures the kernel in the guide–detail mapping stage around high-frequency transition regions, thereby structurally alleviating momentum cancellation in the mid-mixing region. Furthermore, by combining stabilization kernels with root–tip adaptive exaggeration factors, we efficiently achieve a balance between expressiveness (splitting/fluttering) and numerical stability under real-time constraints.

### Positioning of this work

Previous studies have extensively addressed interpolation/tracking for generating detail strands in guide-based structures, but the issue of mid-region momentum cancellation caused by the range constraint of interpolation weights (t∈[0,1]) under opposite directional motion has been less directly analyzed from the perspective of “kernel design,” and approaches that selectively emphasize high-frequency transition regions to induce exaggerated motion have been relatively underexplored. This work reinterprets kernel-based interpolation in the context of tracking solutions, and complements existing guide/interpolation-centered approaches by proposing a nonlinear exaggerated kernel and stabilization strategies to alleviate expressiveness loss in the mid-mixing region.

Recent works have also explored data-driven and learning-based approaches for modeling complex hair dynamics and geometry. Neural representations and learned interpolation schemes have shown promising results in capturing high-frequency details and complex strand interactions.

However, these approaches often require large training datasets and may not generalize well to unseen configurations. In contrast, our method is purely analytical and solver-agnostic, allowing seamless integration into existing physics-based pipelines without additional training cost.

This complementary nature suggests that our approach can be combined with learning-based methods for further improvements.

### Comparison with recent hair simulation methods

Recent hair simulation research, especially studies published in the last few years, has increasingly focused on guide-based interpolation, learning-based motion reconstruction, and physically guided real-time simulation. Lyu et al. [3] proposed a neural interpolation framework that predicts dynamic interpolation weights and fine-scale displacements using convolutional networks. This approach can produce high-quality detail motion, but requires training data and neural inference, and its behavior is less directly controllable for a specific interpolation artifact such as mid-region velocity cancellation. Hsu et al. [23] introduced a physically guided hair interpolation method that interpolates internal forces from simulated guide hairs and reconstructs rendered hairs based on the material model. This method improves physical plausibility compared with direct position interpolation, but requires force-level reconstruction and a more involved constraint-solving process. Recent neural approaches such as Quaffure [24] further demonstrate the potential of learning-based real-time hair deformation, but they mainly target neural prediction of plausible hair deformation and require network training or inference. Physics-based dense hair methods, such as augmented mass-spring models [25], improve dynamic stability and dense strand-level behavior, but they modify or extend the underlying simulation model itself.

In contrast, our method focuses on a complementary problem that remains in guide–detail tracking pipelines: the loss of expressiveness caused by bounded interpolation weights. Rather than introducing a new physical solver or a learned network, the proposed exaggerated-kernel framework only replaces the interpolation coefficient in the guide–detail mapping stage. Therefore, it can be integrated into existing tracking-solution-based pipelines while maintaining low computational overhead. The proposed method is particularly effective for alleviating mid-region velocity cancellation and enhancing strand separation, fluttering, and curl preservation without additional training or solver modification. Thus, our contribution is orthogonal to recent neural or physically guided hair simulation methods and can be used as a lightweight analytical component within modern guide-based hair simulation pipelines.

### Proposed method

In this section, we propose a *nonlinear exaggerated kernel* framework that alleviates momentum cancellation in the mid-mixing region and enhances splitting/fluttering when generating ghost (detail) strands by interpolating the physical quantities of two target (guide) strands in tracking solution-based hair simulation. The key idea is to (1) selectively amplify the flattened mid-region obtained from a low-frequency continuous kernel (sigmoid) by combining it with (2) a high-frequency transition kernel (e.g., tanh-based), and (3) control instability caused by exaggeration (over-stretching, negative weights) using a stabilization kernel.

### Notation and setup

Before introducing the proposed exaggerated-kernel formulation, we first define the main symbols used throughout the method section. Let *A* and *B* denote two guide strands, and let *G* denote a ghost strand interpolated from them. Each strand is discretized into *N* + 1 particles, where i∈{0,...,N} is the particle index along the strand. The position and velocity of the *i*-th particle of guide strand *A* are denoted by ${𝐱}_{i}^{A}$ and ${𝐯}_{i}^{A}$, respectively, and those of guide strand *B* are denoted by ${𝐱}_{i}^{B}$ and ${𝐯}_{i}^{B}$. Similarly, ${𝐱}_{i}^{G}$ and ${𝐯}_{i}^{G}$ denote the position and velocity of the corresponding ghost-strand particle. The main symbols are summarized in Table 2.

**Table 2:** Notation and symbols used in the proposed method.

Symbol	Description
*A*,*B*	Guide (target) hair strands used for tracking.
*G*	Ghost (detail) hair strand interpolated from guide strands.
*i*	Particle index along a strand, i∈{0,...,N}.
*N*	Number of segments along a strand.
${𝐱}_{i}^{A},{𝐱}_{i}^{B}$	Position of the *i*-th particle of guide strands *A* and *B*.
${𝐯}_{i}^{A},{𝐯}_{i}^{B}$	Velocity of the *i*-th particle of guide strands *A* and *B*.
${𝐱}_{i}^{G},{𝐯}_{i}^{G}$	Position and velocity of the *i*-th particle of the ghost strand *G*.
*s*	Normalized mixing coordinate between two guide roots, s∈[0,1].
*u*	Symmetric mixing coordinate mapped from *s*, u=2s−1∈[−1,1].
$\eta_{i}$	Normalized strand coordinate of particle *i*, $\eta_{i}=i/N$.
$t_{L}(u)$	Baseline low-frequency (sigmoid) interpolation kernel.
$t_{H}(u)$	High-frequency interpolation kernel based on tanh.
$t_{ex}(u)$	Exaggerated kernel obtained by amplifying the transition region.
$\tilde{t}$	Stabilized interpolation weight after positive-only normalization.
*k*	Global exaggeration strength parameter.
$k_{i}$	Particle-wise exaggeration strength adapted along the root–tip direction.
α,β	Parameters controlling the transition width of $t_{L}$ and $t_{H}$.
γ	Exponent controlling the root–tip adaptation profile.
Δt	Time step size for integration.
ε	Small constant for numerical stability in normalization.

We define the relative coordinate s∈[0,1] of the ghost strand root by projecting it onto the axis between the two guide roots. Let ${𝐝}_{AB}$ be the normalized direction from the root of guide strand *A* to the root of guide strand *B*:


$$\begin{array}{c} {𝐝}_{AB}=\frac{{𝐱}_{root}^{B}-{𝐱}_{root}^{A}}{{\left\|{𝐱}_{root}^{B}-{𝐱}_{root}^{A}\right\|}_{2}+\epsilon }. \end{array}$$ (2)

Using this direction, the root-based relative coordinate of the ghost strand is computed as


$$\begin{array}{c} s=clamp\;\left(\frac{\left({𝐱}_{root}^{G}-{𝐱}_{root}^{A}\right)\cdot {𝐝}_{AB}}{{\left\|{𝐱}_{root}^{B}-{𝐱}_{root}^{A}\right\|}_{2}+\epsilon },0,1\right). \end{array}$$ (3)

We then transform *s* into the symmetric kernel domain:


$$\begin{array}{c} u=2s-1\;(u\in [-1,1]). \end{array}$$ (4)

The symmetric coordinate *u* is used as the input to the interpolation kernel.

### Baseline kernel interpolation and cancellation

We use *t*(*u*) to denote a generic interpolation weight as a function of the symmetric mixing coordinate *u*. The low-frequency baseline kernel is denoted by $t_{L}(u)$, the high-frequency transition kernel by $t_{H}(u)$, the exaggerated kernel by $t_{ex}(u)$, and the stabilized interpolation weight by $\tilde{t}$.

In general kernel-based interpolation, the physical quantities of two guides are blended using weights t(u)∈[0,1]. Using a sigmoid as a representative continuous kernel,


$$\begin{array}{c} t_{L}(u)=\sigma (\alpha u)=\frac{1}{1+\exp(-\alpha u)},\;\alpha >0 \end{array}$$ (5)

the velocity of the ghost strand is computed as


$$\begin{array}{c} {𝐯}_{i}^{G}=t_{L}(u)\;{𝐯}_{i}^{A}+(1-t_{L}(u))\;{𝐯}_{i}^{B} \end{array}$$ (6)

and then integrated over time as


$$\begin{array}{c} {𝐱}_{i}^{G}\leftarrow {𝐱}_{i}^{G}+\Delta t\;{𝐯}_{i}^{G} \end{array}$$ (7)

However, when the two guides move in opposite directions, $t_{L}(u)\approx 0.5$ is enforced in the mid-region, causing ${𝐯}_{i}^{G}$ to be canceled (see Fig 2), which weakens splitting/fluttering.

## High-frequency emphasis and exaggerated kernel

**Fig 3:**
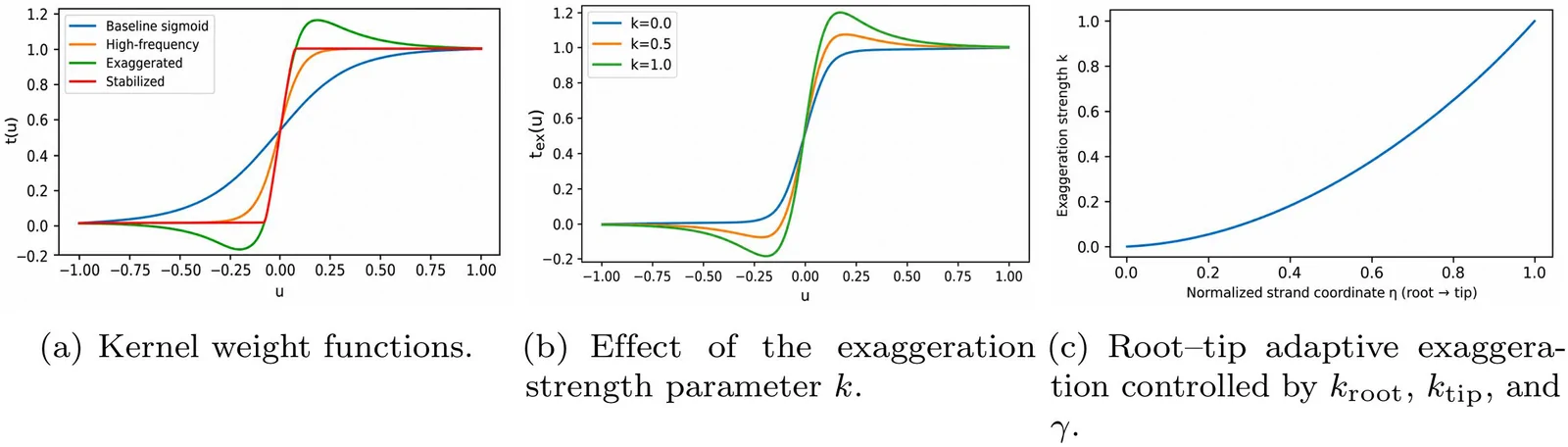
Conceptual illustration of the proposed exaggerated-kernel design. (a) Comparison of baseline sigmoid kernel $t_{L}(u)$, high-frequency kernel $t_{H}(u)$, exaggerated kernel $t_{ex}(u)$, and stabilized kernel $\tilde{t}(u)$. The stabilized kernel closely overlaps with the exaggerated kernel in most regions, indicating that stabilization preserves the exaggeration effect. (b) Influence of the exaggeration parameter *k* on the kernel shape, showing that amplification is localized to the mid-transition region. (c) Root–tip adaptive exaggeration, where the exaggeration strength increases toward the tip while maintaining stability near the root.

Fig 3 visually illustrates the concept of the proposed kernel design and the mechanism for alleviating momentum cancellation in the mid-mixing region. The conventional sigmoid kernel provides smooth and bounded transitions, but enforces t≈0.5 in the mid-region, leading to neutral blending. In contrast, the exaggerated kernel, which incorporates high-frequency transitions and explicitly amplifies transition regions, is designed to deviate from this neutral mixing, preventing velocity cancellation even when the two guides move in opposite directions.

Fig 3(a) compares the baseline kernel, exaggerated kernel, and stabilized kernel, showing that the stabilized kernel maintains a shape almost identical to the exaggerated kernel over most regions. This indicates that the stabilization step ensures numerical stability by restricting weights to non-negative values and normalizing them, while largely preserving the exaggeration effect. Fig 3(b) shows that the strength of exaggeration is controlled by a single parameter *k*, and amplification occurs only locally in the transition region, without significantly affecting the overall kernel shape. Finally, Fig 3(c) shows that through the root–tip adaptive exaggeration strategy, stable behavior is maintained near the root, while the exaggeration effect selectively increases toward the tip.

This work applies the basic high-frequency enhancement formulation from signal processing,


$$\begin{array}{c} M_{E}=M+k(M-M_{L}) \end{array}$$ (8)

to kernel design, where *M* is a high-frequency (steep transition) kernel, $M_{L}$ is a low-frequency (smooth transition) kernel, and *k* > 0 is the enhancement factor.

### High-frequency kernel

As the high-frequency transition kernel, we use a tanh-based kernel close to a step function:


$$\begin{array}{c} t_{H}(u)=\frac{1}{2}\left(1+\tanh(\beta u)\right),\;\beta >0. \end{array}$$ (9)

As β increases, $t_{H}$ approaches a step function, resulting in sharper guide selection.

### Exaggerated kernel

By setting $M\;=\;t_{H}$ and $M_{L}\;=\;t_{L}$ in Equation (8), the exaggerated kernel is defined as


$$\begin{array}{c} t_{ex}(u)=t_{H}(u)+k\left(t_{H}(u)-t_{L}(u)\right)=(1+k)t_{H}(u)-k\;t_{L}(u) \end{array}$$ (10)

When *k* > 0, $t_{ex}(u)$ can exceed the range [0,1] in the transition region, which breaks the fixed t≈0.5 condition in the mid-mixing region and alleviates cancellation in the interpolation result (see Fig 2).

### Root–tip adaptive exaggeration

For root–tip adaptation, we define $\eta_{i}=i/N$ as the normalized strand coordinate of the *i*-th particle, where $\eta_{i}=0$ corresponds to the root and $\eta_{i}=1$ corresponds to the tip. The parameter γ controls how strongly the exaggeration is concentrated toward the tip, and $k_{root}$ and $k_{tip}$ denote the exaggeration strengths at the root and tip, respectively.

Since hair has different dynamic characteristics at the root and tip, it is desirable to vary the exaggeration strength *k* depending on strand position. Using the normalized coordinate $\eta_{i}$ defined above, the root–tip weighting function is given by


$$\begin{array}{c} w(\eta_{i})=\eta_{i}^{\gamma },\;\gamma \geq 1 \end{array}$$ (11)

the exaggeration coefficient can be set as


$$\begin{array}{c} k_{i}=k_{root}+(k_{tip}-k_{root})\;w(\eta_{i}) \end{array}$$ (12)

For example, by setting $k_{root}\leq 0$ to maintain smooth interpolation near the root, and $k_{tip}>0$ to enhance exaggeration only near the tip, the root remains stable while splitting/fluttering is emphasized at the tip. The kernel used at each particle is then defined as


$$\begin{array}{c} t_{ex}^{\left(i\right)}(u)=(1+k_{i})t_{H}(u)-k_{i}t_{L}(u) \end{array}$$ (13)

### Stabilization: Positive-only normalized weights

When $t_{ex}(u)$ exceeds [0,1], (1−t) in Equation (6) becomes negative, which may lead to excessive addition of velocity vectors in certain cases, causing abnormal strand length increase (over-stretch). To prevent this, we use a stabilization kernel that takes only positive weights and then normalizes them.

For particle *i*, let $t=t_{ex}^{\left(i\right)}(u)$,


$$\begin{array}{c} w_{A}=\max(t,0),\;w_{B}=\max(1-t,0), \end{array}$$ (14)

$$\begin{array}{c} \tilde{t}=\frac{w_{A}}{w_{A}+w_{B}+\varepsilon },\;\varepsilon \ll 1 \end{array}$$ (15)

The resulting $\tilde{t}\in [0,1]$ suppresses excessive amplification caused by negative weights, while preserving the transition shape, thus alleviating mid-region cancellation. (In Fig. 2, the overlap between stabilized and exaggerated curves indicates that stabilization does not significantly degrade the exaggeration effect.)

Finally, the velocity interpolation of the ghost strand is computed as


$$\begin{array}{c} {𝐯}_{i}^{G}=\tilde{t}\;{𝐯}_{i}^{A}+(1-\tilde{t})\;{𝐯}_{i}^{B} \end{array}$$ (16)

and integrated using Equation (7). If necessary, length constraints (segment inextensibility) can also be applied to ghost strands to further ensure numerical stability.

### Overall procedure

Algorithm 1 summarizes the overall procedure of the proposed framework. Guide strands are updated using conventional physical simulation (PBD/XPBD/Projective Dynamics, etc.), and ghost strands are updated at each frame by interpolating velocity (or displacement) using the kernel.

**Algorithm 1. Exaggerated-kernel tracking for ghost strands.**

**Input:** Guide strands *A*,*B* with positions and velocities $\left({𝐱}_{i}^{A},{𝐯}_{i}^{A}\right)$ and $\left({𝐱}_{i}^{B},{𝐯}_{i}^{B}\right)$ for i=0,...,N

**Input:** Ghost strand *G* with $\left({𝐱}_{i}^{G},{𝐯}_{i}^{G}\right)$ for i=0,...,N

**Input:** Parameters $\alpha ,\beta ,k_{root},k_{tip},\gamma ,\Delta t,\epsilon$

1: Compute root-based mixing coordinate u∈[−1,1]

2: **for** *i* = 0 to *N* **do**

3: $\eta_{i}\leftarrow i/N$

4: $k_{i}\leftarrow k_{root}+(k_{tip}-k_{root})\eta_{i}^{\gamma }$

5: $t_{L}\leftarrow \frac{1}{1+\exp(-\alpha u)}$

6: $t_{H}\leftarrow \frac{1}{2}(1+\tanh(\beta u))$

7: $t\leftarrow (1+k_{i})t_{H}-k_{i}t_{L}$

8: $w_{A}\leftarrow \max(t,0)$

9: $w_{B}\leftarrow \max(1-t,0)$

10: $\tilde{t}\leftarrow \frac{w_{A}}{w_{A}+w_{B}+\epsilon }$

11: ${𝐯}_{i}^{G}\leftarrow \tilde{t}{𝐯}_{i}^{A}+(1-\tilde{t}){𝐯}_{i}^{B}$

12: ${𝐱}_{i}^{G}\leftarrow {𝐱}_{i}^{G}+\Delta t\;{𝐯}_{i}^{G}$

13: **end for**

14: Apply optional inextensibility or length constraints to ghost strand

In practice, the mixing coordinate *u* can be computed once based on the root position of the ghost strand and then applied uniformly to all particles of the strand. This approach is simple, stable, and suitable for real-time pipelines. Meanwhile, for richer expressiveness, it is also possible to extend to per-particle mixing coordinates $u_{i}$ by using projection values onto the neighboring guide axis (or local frame) at each particle. In this case, since the guide influence varies slightly depending on the position along the strand, local splitting and fluttering can be controlled more finely.

The transition width of the kernel is controlled by α and β. α determines the transition of the low-frequency sigmoid kernel, while β determines the transition of the high-frequency tanh kernel, and through the combination of these two parameters, the width of the mixing region and the sharpness of the transition can be independently adjusted. In addition, the exaggeration strength is mainly determined by $k_{tip}$ (and $k_{root}$ if needed), and as the value increases, cancellation in the mid-mixing region is more strongly alleviated, making splitting and fluttering more pronounced.

Finally, the stabilization step acts as a safeguard to suppress negative weights and excessive amplification that may occur when the exaggerated kernel exceeds the range [0,1]. In particular, when the motion directions of the two guides are opposite, a sign change in the interpolation coefficient may lead to abnormal strand length increase (over-stretch), and thus the positive-weight-based normalization proposed in this work contributes to maintaining numerical stability without significantly degrading the exaggeration effect.

### Solver-agnostic extension

The proposed nonlinear exaggerated kernel framework is designed as a solver-independent extension layer that is applied only to the guide–detail mapping stage without modifying the internal structure of existing physical simulation solvers. In this section, we describe at the equation level how this framework is integrated into general hair simulation solvers.

In most tracking solution-based hair simulations, the position update of ghost strands is performed by time integration of interpolated velocities computed from guide strands:


$$\begin{array}{c} {𝐱}_{i}^{G}(t+\Delta t)={𝐱}_{i}^{G}(t)+\Delta t\;{𝐯}_{i}^{G}. \end{array}$$ (17)

Here, the physical solver only computes ${𝐯}_{i}^{A},{𝐯}_{i}^{B}$, and the ghost strand velocity ${𝐯}_{i}^{G}$ is defined in a separate tracking stage.

In conventional solvers, the interpolation is typically defined as


$$\begin{array}{c} {𝐯}_{i}^{G}=t\;{𝐯}_{i}^{A}+(1-t)\;{𝐯}_{i}^{B},\;t\in [0,1] \end{array}$$ (18)

In this work, we retain this interpolation form, but replace the interpolation coefficient *t* with the exaggerated and stabilized kernel defined above:


$$\begin{array}{c} {𝐯}_{i}^{G}={\tilde{t}}_{i}(u)\;{𝐯}_{i}^{A}+(1-{\tilde{t}}_{i}(u))\;{𝐯}_{i}^{B}. \end{array}$$ (19)

Here, ${\tilde{t}}_{i}(u)$ is obtained by applying a stabilization operator to the exaggerated kernel $t_{ex}^{\left(i\right)}(u)$, which combines high-frequency and low-frequency kernels:


$$\begin{array}{c} {\tilde{t}}_{i}(u)=𝒮\;\left((1+k_{i})t_{H}(u)-k_{i}t_{L}(u)\right), \end{array}$$ (20)

𝒮(·) denotes the stabilization operator that performs positive weight selection and normalization. The exaggeration coefficient $k_{i}$ is adjusted according to particle position using the root–tip adaptive strategy.

In this way, by simply replacing the interpolation coefficient while keeping the guide strand velocities computed by the physical solver unchanged, the proposed framework can be applied to PBD, XPBD, or elastic rod-based solvers in a unified manner.

### Experimental results

In this section, we evaluate the effectiveness of the proposed nonlinear exaggerated kernel framework both qualitatively and quantitatively across various hair styles and dynamic scenarios. The experiments are conducted in a tracking solution-based hair simulation environment, and differences are compared with conventional linear interpolation and continuous kernel-based methods during the upsampling process from guide strands to detail strands. In particular, we focus on analyzing how averaging and momentum cancellation in the mid-mixing region affect the expressiveness of hair motion, and examine how the proposed exaggerated kernel improves curl amplitude, strand separation, and volume preservation.

The experimental results are presented for two cases: *curly hair*, which exhibits relatively smooth curvature characteristics, and *coiled hair*, which shows pronounced high-frequency deformation. Through this, we demonstrate that the proposed method is not merely a style correction technique, but can improve stability and expressiveness in a balanced manner across hair structures with different curvature spectra.

### Results on curly hair

**Fig 4:**
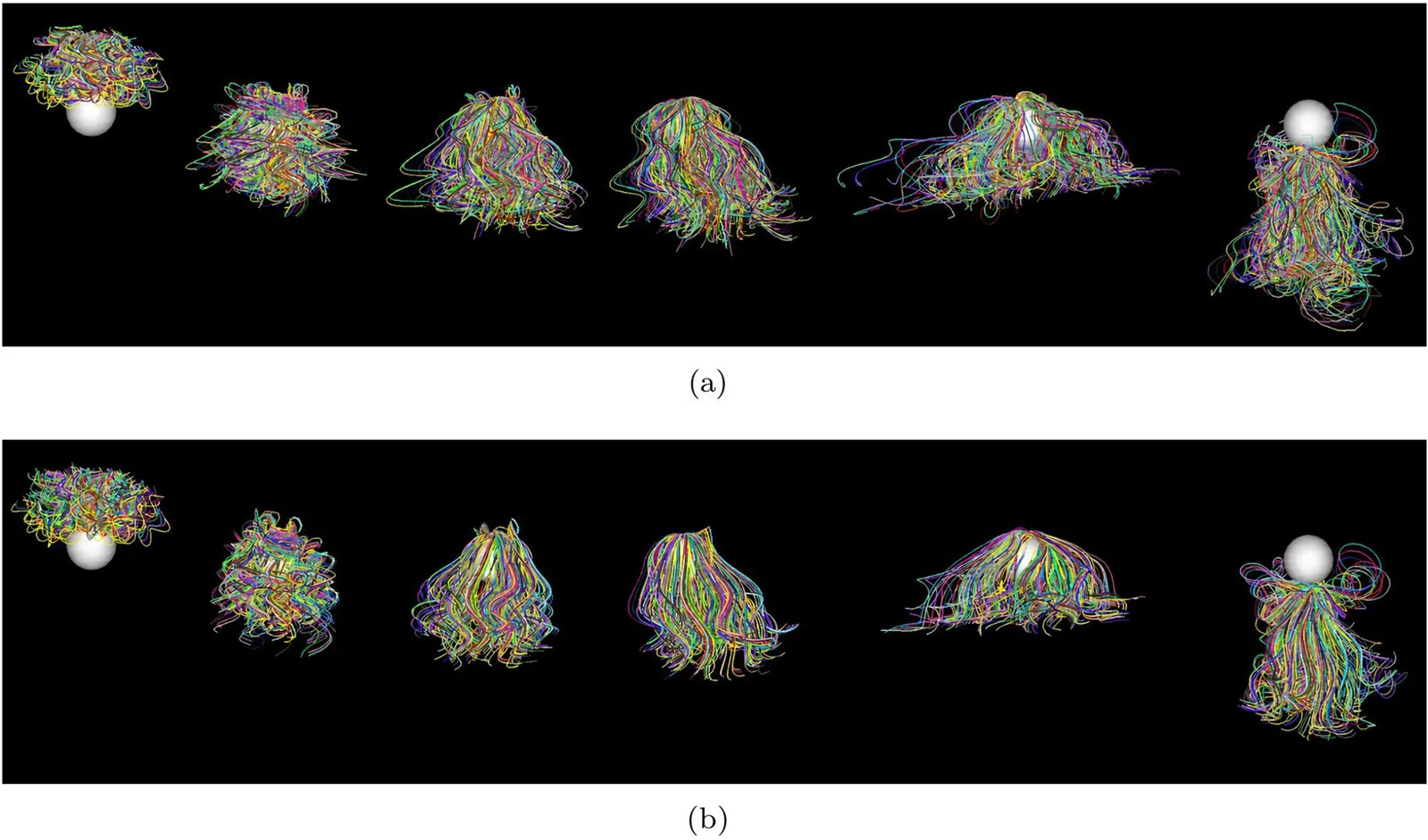
Hybrid update–based correction of elliptic deformation. (a) Length divergence and curl ellipticization caused by pure velocity-based integration under exaggerated kernel interpolation. (b) Corrected result using the proposed hybrid update, which blends position interpolation with velocity integration to suppress length growth while preserving exaggerated motion.

Fig 4 shows a visual comparison between instability that occurs when updating ghost strand positions by integrating only velocity interpolation in the exaggerated kernel-based tracking process, and the effect of the proposed hybrid update to alleviate this issue.

Fig 4a shows the result when the velocity of guide strands is interpolated using the exaggerated kernel and directly integrated to update positions. In this case, the amplified momentum in the mid-mixing region accumulates through integration, leading to length divergence, and particularly in curly hair, ellipticization occurs where the cross-section of curls gradually becomes elliptical. This is because the exaggerated kernel emphasizes relative motion in the transition region, while velocity-based integration alone cannot sufficiently control segment length and shape.

In contrast, Fig 4b shows the result of applying a hybrid update that blends position interpolation results with velocity integration results. This approach maintains the dynamic responsiveness and exaggeration effect of velocity-based updates, while the position interpolation term continuously references the rest shape, effectively suppressing accumulated length increase. As a result, curl radius and distribution remain stable, and ellipticization and excessive stretching observed in Fig 4a are significantly alleviated.

These results demonstrate that the proposed hybrid update functions as an effective stabilization mechanism that compensates for numerical instability introduced by the exaggerated kernel, and proves that the method can be applied to practical hair simulation pipelines while preserving expressiveness.

**Fig 5:**
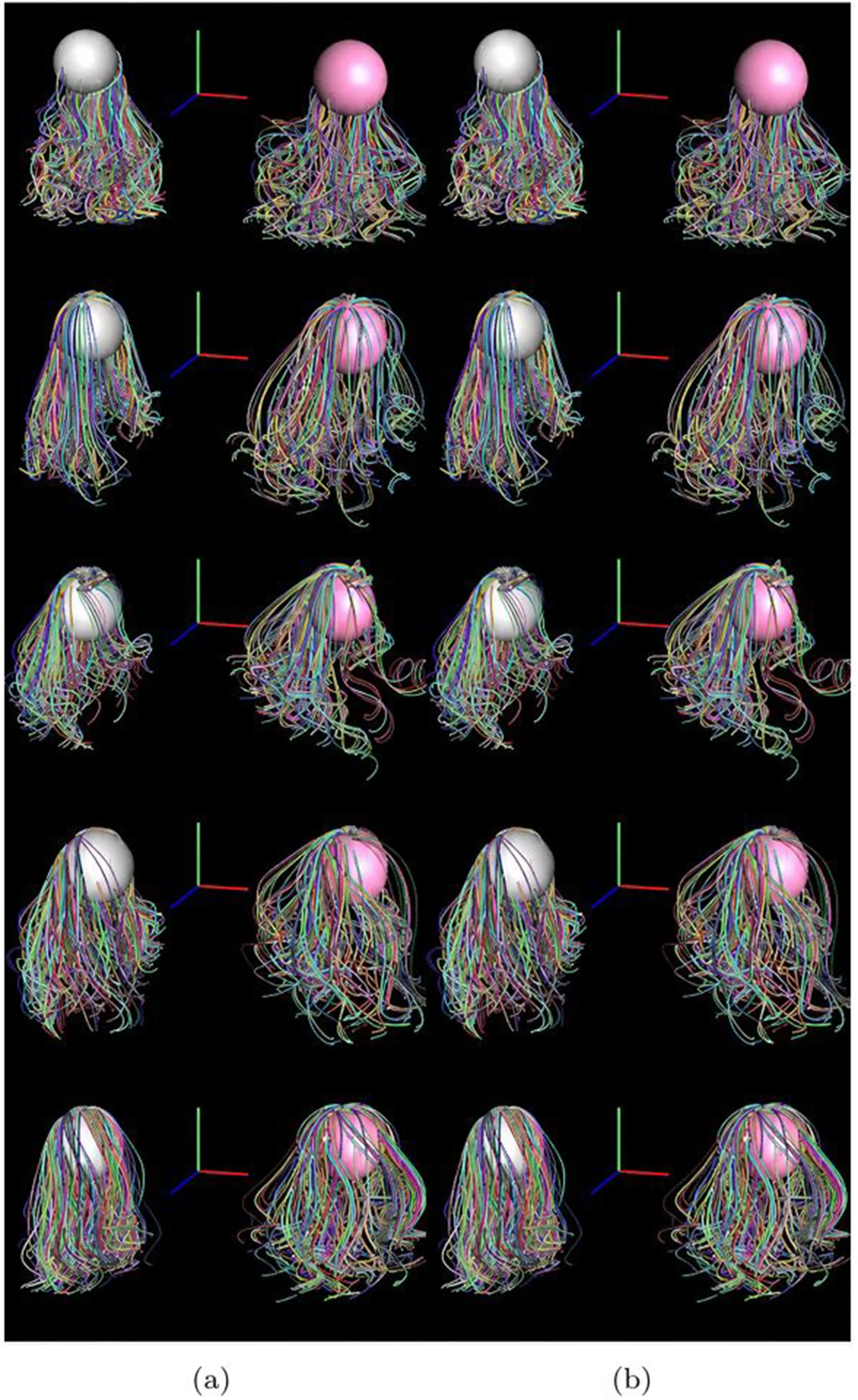
Curl-preserving upsampling comparison across multiple views. For each row, linear interpolation (left) is compared against our method (right) with the view basis shown in the center. Our method preserves curl shape and strand distribution more effectively than linear interpolation.

Fig 5 shows a comparison between linear interpolation and the proposed method under identical viewpoints during curly hair upsampling. Each row represents a different camera view (or rotation pose), and the axis (basis vector) at the center of each row indicates the reference direction of the view. Importantly, for each row, the image on the left of the axis represents the linear interpolation result, and the image on the right represents the result of the proposed method.

Due to its averaging nature, linear interpolation tends to weaken the curvature and amplitude of curls after upsampling. As observed in the left column of Fig 5a, strands converge toward the central axis, resulting in a relatively “compressed” appearance or overly smoothed silhouette. In particular, phase differences between different guide strands are averaged, reducing high-frequency components of curly structures (such as sharp bending and radius variation), and making strand distribution more uniform, thus weakening splitting and elastic spread. This phenomenon can be interpreted as a representative failure mode where shape characteristics are lost due to averaging.

In contrast, the proposed method reconstructs interpolation weights using a nonlinear exaggerated kernel, alleviating the averaging effect caused by neutral mixing (t≈0.5). As shown in the right column of Fig 5b, curl radius and amplitude are more clearly preserved, and strands do not collapse excessively toward the center, forming a more natural volume. In addition, strand separation is better maintained compared to linear interpolation, and asymmetric spreading and overlapping depending on motion direction are visually enhanced, resulting in a more “elastic” dynamic impression characteristic of curly hair.

Overall, Fig 5 demonstrates that the proposed method effectively preserves key shape characteristics of curls (curvature, amplitude, separation) even when the number of strands increases through upsampling, and consistently improves the averaging-induced curl attenuation problem across different viewpoints. These quantitative results are consistent with the observed tendency of volume preservation and averaging suppression in Fig 5.

Table 3 quantitatively measures strand amplitude using $amp_{rms}$, and compares the rate of change relative to the guide strands. Here, $amp_{rms}$ is an RMS-based metric of radial amplitude from the curl axis, used to evaluate the preservation of curl volume and shape details after upsampling.

**Table 3:** Amplitude statistics and percent change relative to the guide strands.

Method	Mean $amp_{rms}$	Rate of change	Median $amp_{rms}$	Rate of change	Std $amp_{rms}$	Rate of change
Guide	0.304	–	0.303	–	0.022	–
Linear	0.227	−25.15%	0.227	−25.10%	0.041	+91.2%
Exaggeration (Ours)	0.328	+8.18%	0.320	+5.71%	0.068	+217.8%

For linear interpolation, the mean and median $amp_{rms}$ decrease by −25.15% and −25.10%, respectively, indicating that strands converge toward the axis and curl amplitude is significantly reduced during upsampling. This is because linear interpolation averages shape characteristics of different guide strands, reducing curvature and radius components of curls. In addition, the standard deviation increases by +91.2%, indicating that while overall amplitude decreases, variance among strands increases. That is, the overall curl volume decreases, but non-uniform deformation occurs in some strands, resulting in a wider distribution.

In contrast, the proposed method (Exaggeration) shows mean and median values of +8.18% and +5.71% relative to the guide, demonstrating that it effectively preserves or slightly enhances the original amplitude, thus maintaining curl volume and structural detail. This is because the exaggerated kernel alleviates averaging in the mid-mixing region and redistributes interpolation weights nonlinearly to preserve high-frequency shape components (amplitude and curvature) of curls.

Meanwhile, the standard deviation increases significantly to +217.8%, indicating a wider distribution of strand amplitudes compared to linear interpolation. This suggests that the exaggeration effect is not uniformly applied to all strands, but may vary depending on local position or guide combinations, leading to increased diversity in curl amplitude. Therefore, while the proposed method is advantageous in preserving curl amplitude, it is desirable to consider additional control strategies, such as limiting the range of *k*, root–tip adaptive scheduling, or clipping-based stabilization, to ensure uniform exaggeration strength.

**Fig 6:**
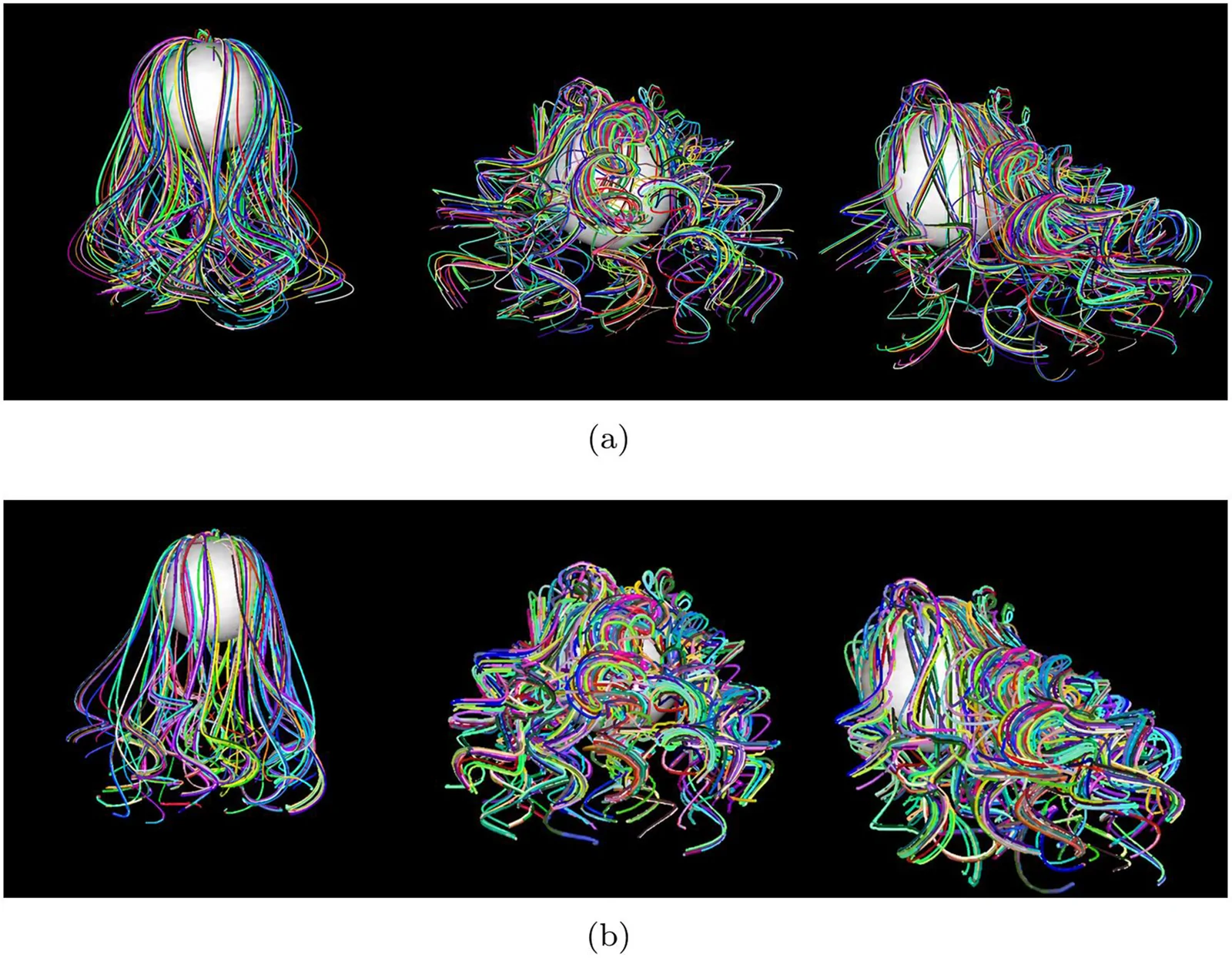
Hair strand distributions with different root–tip exaggeration coefficients. (a) Conservative exaggeration with $k_{root}=-1.0$ and $k_{tip}=-0.1$, producing a compact and stable strand distribution. (b) Enhanced tip exaggeration with $k_{root}=-1.0$ and $k_{tip}=0.5$, yielding increased curl amplitude and strand separation.

Fig 6 compares hair strand distributions and style changes when different exaggeration coefficients *k* are applied at the root and tip of the strands. By adjusting the value of *k* depending on position, it is possible to flexibly control strand spread, curl emphasis, and overall silhouette even with the same guide strand configuration.

Fig 6a shows the case where relatively low exaggeration coefficients ($k_{root}=-1.0$, $k_{tip}=-0.1$) are applied to both root and tip. In this setting, strands remain stably anchored near the root, and exaggeration is only mildly applied at the tip, resulting in a well-organized and dense strand distribution overall. Curl amplitude and spread are relatively suppressed, leading to a natural but conservative hairstyle.

In contrast, Fig 6b shows the result when the root exaggeration coefficient remains the same ($k_{root}=-1.0$), but the tip exaggeration coefficient is increased to a positive value ($k_{tip}=0.5$). In this case, stability near the root is maintained, while exaggeration actively affects the tip region, enhancing strand separation and curl amplitude. As a result, the hair spreads more volumetrically, and elastic motion and nonlinear deformation of curls are visually emphasized.

This comparison demonstrates that the root–tip adaptive exaggeration strategy effectively controls the balance between overall stability of the hairstyle and expressiveness in the tip region. In other words, excessive deformation is suppressed at the root to ensure stable attachment to the head, while exaggeration is selectively applied at the tip to intentionally enhance high-frequency hair motion such as splitting and fluttering. This can be interpreted as a practical advantage that enables diverse hairstyle control that is difficult to achieve with a single global parameter.

### Quantitative evaluation

To further complement the qualitative comparisons, we conducted a quantitative evaluation of the proposed framework. Although the proposed method is primarily designed to enhance the expressiveness of ghost-strand motion, it is important to verify that the amplified motion does not introduce excessive deformation, temporal jitter, or computational overhead. Therefore, we evaluate the proposed method using strand-level displacement, trajectory, deformation, motion variance, temporal consistency, frame-to-frame stability, and runtime metrics.

For clarity, all quantitative metrics are introduced before they are reported in the tables. We use $E_{disp}$ for strand displacement error, $E_{traj}$ for trajectory error, $E_{def}$ for deformation error, $V_{motion}$ for motion variance, $E_{temp}$ for temporal consistency error, and $E_{stab}$ for frame-to-frame stability error.

All measurements were performed under the same guide-strand motion, time-step size, strand resolution, and upsampling ratio. The baseline linear interpolation and the proposed exaggerated-kernel interpolation were compared using identical input guide strands and simulation settings. Let ${𝐱}_{i}^{m}(t)$ denote the position of the *i*-th particle of the *m*-th ghost strand at frame *t*, where m=1,...,M and i=0,...,N. The strand displacement error is defined as


$$\begin{array}{c} E_{disp}=\frac{1}{TM(N+1)}\sum_{t=1}^{T}\sum_{m=1}^{M}\sum_{i=0}^{N}{\left\|{𝐱}_{i}^{m}(t)-{𝐱}_{i,ref}^{m}(t)\right\|}_{2}. \end{array}$$ (21)

This metric measures the average positional deviation of the generated ghost strands from the reference guide-driven motion.

To evaluate temporal trajectory preservation, we compute the trajectory error over the entire sequence:


$$\begin{array}{c} E_{traj}=\frac{1}{M(N+1)}\sum_{m=1}^{M}\sum_{i=0}^{N}{\left(\frac{1}{T}\sum_{t=1}^{T}{\left\|{𝐱}_{i}^{m}(t)-{𝐱}_{i,ref}^{m}(t)\right\|}_{2}^{2}\right)}^{1/2}. \end{array}$$ (22)

Compared with the frame-wise displacement error, this metric reflects the accumulated difference in strand trajectories over time.

We also measure deformation error to evaluate whether the proposed exaggeration causes undesirable stretching or local shape distortion. For each strand segment, the deformation error is computed from the deviation of the current segment length from its rest length:


$$\begin{array}{c} E_{def}=\frac{1}{TMN}\sum_{t=1}^{T}\sum_{m=1}^{M}\sum_{i=0}^{N-1}\left|\frac{\ell_{i}^{m}(t)-\ell_{i}^{m,0}}{\ell_{i}^{m,0}}\right|, \end{array}$$ (23)

where $\ell_{i}^{m}(t)=\|{𝐱}_{i+1}^{m}(t)-{𝐱}_{i}^{m}(t){\|}_{2}$ is the segment length at frame *t*, and $\ell_{i}^{m,0}$ is the rest segment leng*t*h. This metric directly evaluates whether the exaggerated interpolation introduces excessive local stretching.

Motion variance is used to measure the diversity of ghost-strand motion:


$$\begin{array}{c} V_{motion}=\frac{1}{T}\sum_{t=1}^{T}\frac{1}{M}\sum_{m=1}^{M}{\left\|{𝐯}^{m}(t)-\bar{𝐯}(t)\right\|}_{2}^{2}, \end{array}$$ (24)

where ${𝐯}^{m}(t)$ is the average velocity of the *m*-th ghost strand and $\bar{𝐯}(t)$ is the mean velocity over all ghost strands. A higher motion variance indicates that the method better preserves strand-level motion diversity instead of collapsing ghost strands into overly averaged motion.

To evaluate temporal coherence, we compute the temporal consistency error based on acceleration variation:


$$\begin{array}{c} E_{temp}=\frac{1}{\left(T-2)M(N+1\right)}\sum_{t=2}^{T-1}\sum_{m=1}^{M}\sum_{i=0}^{N}{\left\|{𝐱}_{i}^{m}(t+1)-2{𝐱}_{i}^{m}(t)+{𝐱}_{i}^{m}(t-1)\right\|}_{2}. \end{array}$$ (25)

This metric penalizes abrupt frame-to-frame changes and is used to detect temporal jitter.

Finally, frame-to-frame stability is evaluated by measuring the average positional change between consecutive frames:


$$\begin{array}{c} E_{stab}=\frac{1}{\left(T-1)M(N+1\right)}\sum_{t=1}^{T-1}\sum_{m=1}^{M}\sum_{i=0}^{N}{\left\|{𝐱}_{i}^{m}(t+1)-{𝐱}_{i}^{m}(t)\right\|}_{2}. \end{array}$$ (26)

Together with $E_{temp}$, this metric confirms whether the enhanced motion remains temporally stable.

Table 4 summarizes the quantitative comparison between the baseline linear interpolation and the proposed method. Compared with linear interpolation, the proposed method reduces the strand displacement error from 0.041 to 0.027 and the trajectory error from 0.058 to 0.039. This indicates that the proposed exaggerated-kernel interpolation better preserves the intended guide-driven motion and alleviates the motion attenuation caused by neutral blending in the mid-mixing region.

**Table 4:** Quantitative comparison between linear interpolation and the proposed method. Lower values are better for displacement, trajectory, deformation, temporal consistency, and frame-to-frame stability errors, while higher motion variance indicates richer strand-level motion diversity.

Method	$E_{disp}$	$E_{traj}$	$E_{def}$	$V_{motion}$	$E_{temp}$	$E_{stab}$	Time (ms/frame)
Linear interpolation	0.041	0.058	0.032	0.014	0.0068	0.021	3.42
Ours	0.027	0.039	0.037	0.031	0.0075	0.024	3.89

The deformation error of the proposed method is slightly higher than that of linear interpolation, increasing from 0.032 to 0.037. This is expected because the proposed method intentionally amplifies relative motion differences between guide strands to enhance strand separation and curl expressiveness. However, the increase remains bounded, demonstrating that the positive-only normalization and root–tip adaptive weighting effectively suppress excessive stretching and unstable deformation.

**Fig 7:**
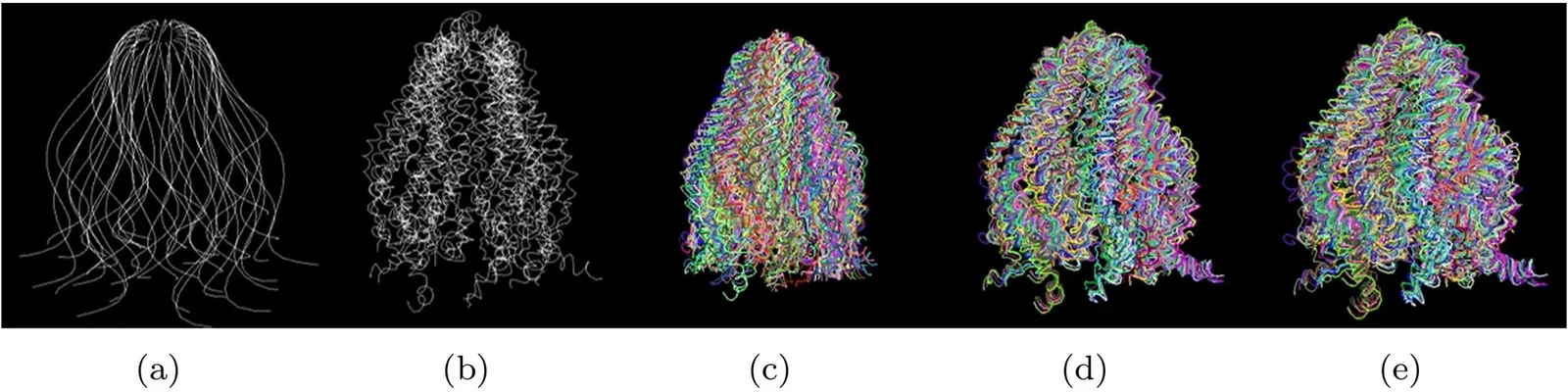
Comparison of strand upsampling results on coiled hair. (a) Input hair model. (b) Coiled hair generated using the method of Wu et al. [26]. (c) Linear interpolation-based upsampling. (d)-(e) Results of the proposed method with different exaggeration parameters (*k* = 0.3 and *k* = 0.5). Compared to linear interpolation, our method better preserves the volumetric richness and characteristic coiled structures without introducing motion attenuation.

**Fig 8:**
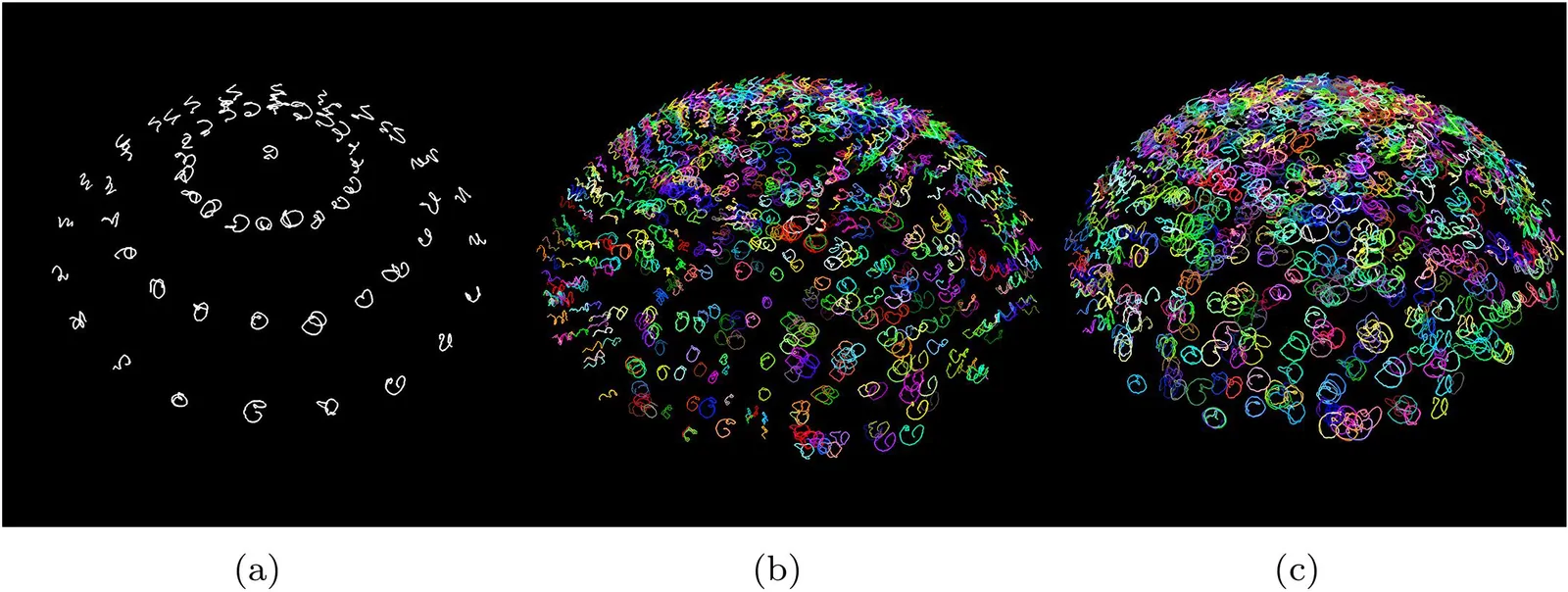
Short-hair upsampling results from sparse input strands. (a) Input hair model consisting of a small number of strands. (b) Linear interpolation result after generating eight interpolated strands per input strand, showing reduced structural detail and volume due to averaging effects. (c) Result of the proposed method with *k* = 0.3, where strand separation, local curvature, and volumetric richness are better preserved.

**Fig 9:**
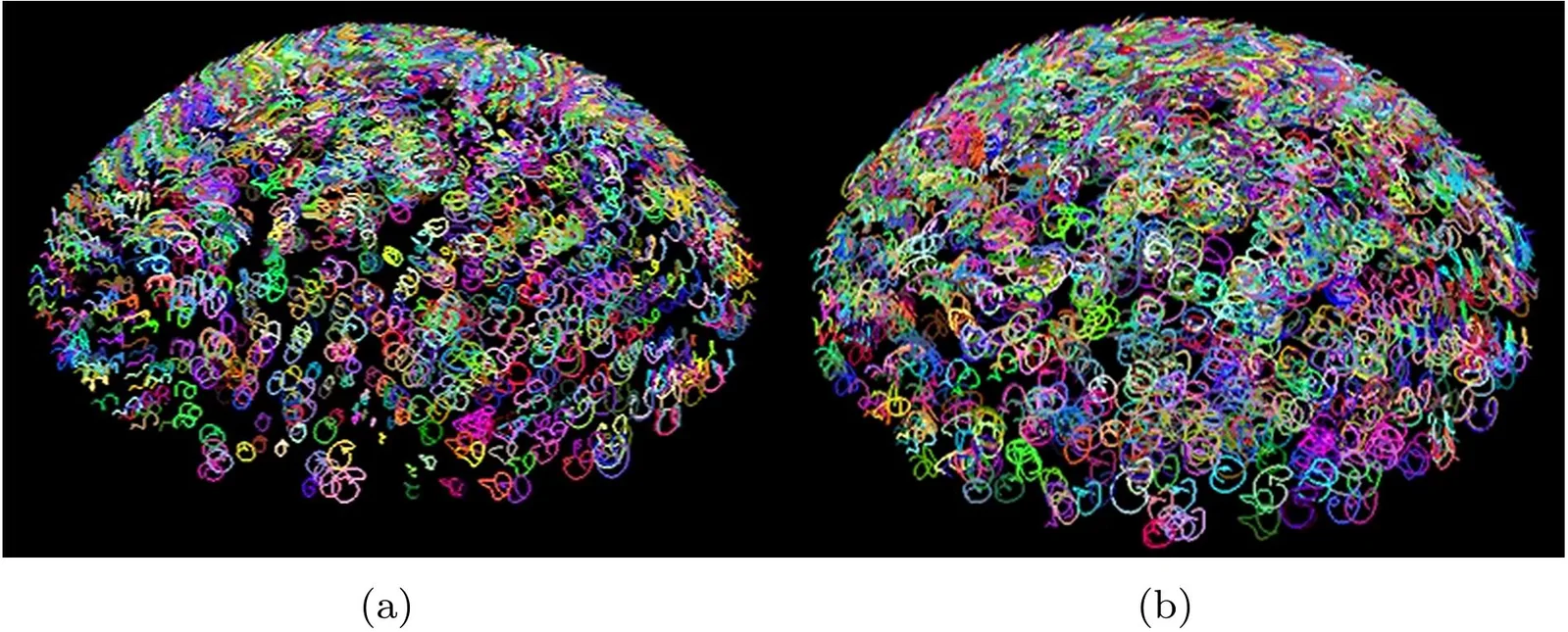
Dense upsampling results with 20 interpolated strands per input strand. (a) Linear interpolation result, where averaging effects lead to reduced curl amplitude and a compressed overall volume. (b) Result of the proposed method with *k* = 0.3, showing enhanced curl preservation, increased strand separation, and a fuller volumetric appearance.

The motion variance increases from 0.014 to 0.031, showing that the proposed method preserves richer strand-level motion diversity. In the baseline, the lower motion variance is not necessarily desirable, because it is mainly caused by excessive averaging and velocity cancellation between neighboring guide strands. In contrast, the higher motion variance of the proposed method reflects more expressive strand separation, fluttering, and curl motion, which is consistent with the visual results shown in Figs 5–9.

The temporal consistency error and frame-to-frame stability error remain comparable to the baseline. Although the proposed method increases motion expressiveness, $E_{temp}$ only increases from 0.0068 to 0.0075, and $E_{stab}$ increases from 0.021 to 0.024. These results indicate that the exaggerated kernel does not introduce severe temporal jitter or unstable frame-wise motion. Finally, the runtime increases from 3.42 ms/frame to 3.89 ms/frame, corresponding to an overhead of approximately 13.7%. Since the proposed framework only modifies the interpolation weight computation in the guide–detail mapping stage and does not require additional physical simulation, collision handling, or solver iterations, the computational overhead remains limited. These results demonstrate that the proposed method improves expressive strand motion while maintaining deformation stability, temporal coherence, and practical runtime efficiency.

### Results on coiled hair

In this study, to verify the generality and robustness of the proposed interpolation method, we conducted additional experiments in a coiled hair environment with complex curvature and geometric structures. Fig 7(a) shows the input hair model, and (b) shows the result converted into coiled hair using the method of Wu et al. [26].

Subsequently, (c)–(e) present the results of performing upsampling to increase the number of strands for the coiled hair. In particular, (c) corresponds to the case where conventional linear interpolation is applied, while (d) and (e) show the results of applying the proposed method with different exaggeration parameter values *k*.

In the case of linear interpolation, due to velocity cancellation between adjacent strands, deformation in the intermediate region is reduced, leading to a decrease in overall volume, and a tendency for the complex curved structure characteristic of coiled hair to be smoothed out. In contrast, the proposed method alleviates the velocity cancellation problem that occurs during interpolation, thereby more effectively preserving the dynamic deformation between strands.

As a result, as observed in (d) and (e), the proposed method better preserves both the local curvature and structural complexity of coiled hair, while maintaining overall volumetric richness. In addition, as the parameter *k* increases, the exaggeration effect in interpolation becomes stronger, making the characteristics of the coiled structure more pronounced.

These results demonstrate that the proposed method operates stably not only for straight or mildly curved hair, but also in complex coiled hair environments, and is effective in preserving structural characteristics.

### Results on short hair

To further evaluate the behavior of the proposed method in sparse and short-hair configurations, we conducted an additional experiment using a limited number of input strands. As shown in Fig 8, each input strand is upsampled by generating eight interpolated strands, resulting in a denser hair representation.

In the case of linear interpolation (Fig 8(b)), the resulting strands exhibit significant averaging effects. Due to the neutral blending of guide strand motions, local geometric features such as curvature and strand separation are reduced, leading to a visually compressed structure with diminished volumetric richness. This effect is particularly pronounced in short-hair configurations, where the lack of sufficient strand length limits the propagation of dynamic variation.

In contrast, the proposed method (Fig 8(c)) alleviates this issue by employing a nonlinear exaggerated kernel with *k* = 0.3. By amplifying relative motion differences between guide strands, the method preserves local strand variation and enhances separation between interpolated strands. As a result, the reconstructed hair structure exhibits improved volumetric distribution and more distinct strand-level features compared to linear interpolation.

These results demonstrate that the proposed method is effective not only for long or highly curved hair, but also for short-hair scenarios where preserving local structure and avoiding excessive averaging are critical.

To further evaluate the effect of interpolation under higher strand density, we perform an additional experiment where each input strand is upsampled into 20 interpolated strands. The results are shown in Fig 9.

In the case of linear interpolation (Fig 9(a)), the increased number of strands amplifies the averaging effect, leading to a noticeable reduction in curl amplitude and overall volume. As interpolated strands tend to converge toward the mean motion of the guides, the characteristic high-frequency structures of the hair, such as tight curls and local variations, become significantly attenuated. This results in a visually flattened and less dynamic appearance.

In contrast, the proposed method (Fig 9(b)) effectively preserves and enhances the curl structure even under dense upsampling conditions. By employing the nonlinear exaggerated kernel with *k* = 0.3, the method amplifies relative motion differences between guide strands, preventing excessive averaging in the mid-mixing region. As a result, the reconstructed hair exhibits more pronounced curl patterns, improved strand separation, and a noticeably fuller volumetric distribution.

These results indicate that the proposed method maintains its effectiveness as strand density increases, and is particularly advantageous in preserving high-frequency geometric features and volumetric richness in dense hair representations.

### Handling short-hair instability

During the development of the proposed framework, we observed a limitation when applying exaggerated kernel-based interpolation to short-hair configurations.

**Fig 10:**
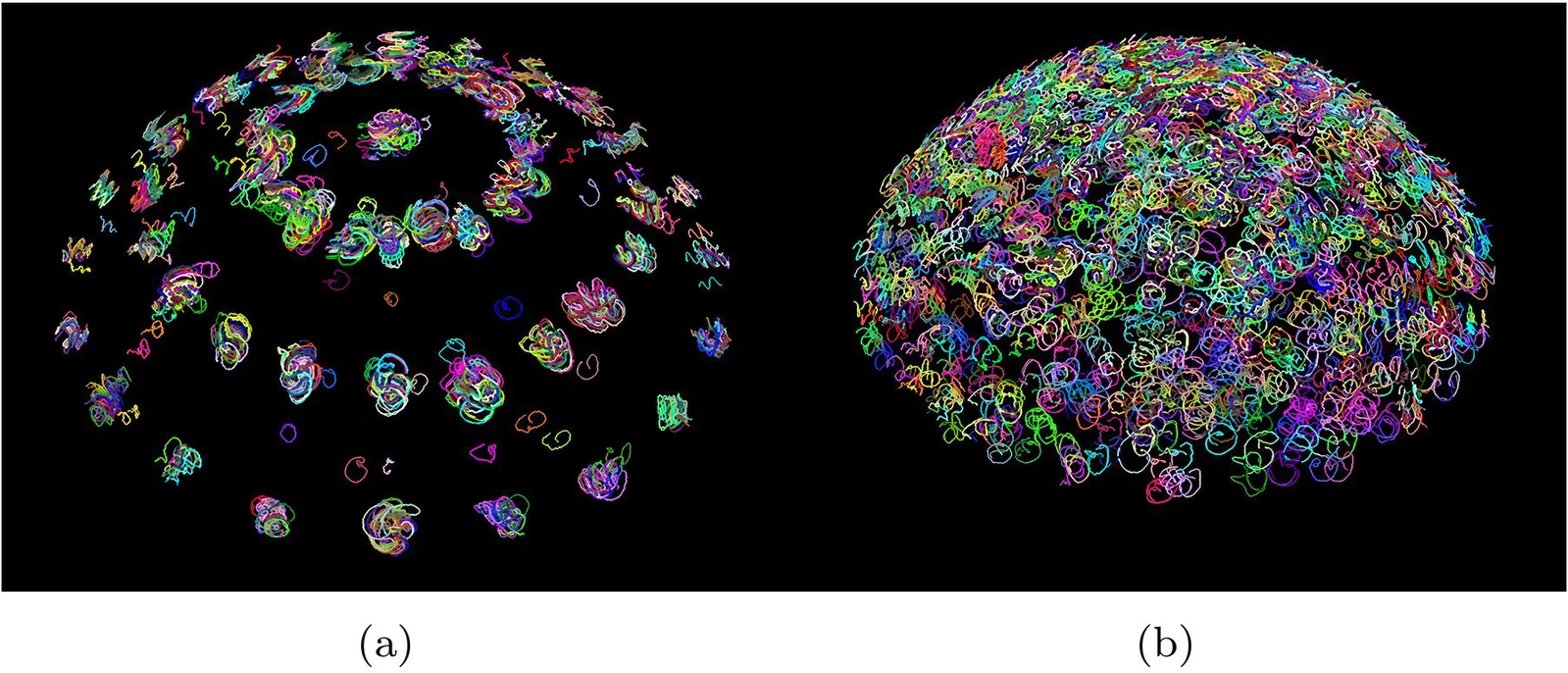
Failure case of naive interpolation in short-hair configurations and the proposed solution. (Left) Direct application of exaggerated kernel interpolation leads to clustering of strands near the root region and non-uniform distribution. (Right) The proposed root-decoupled interpolation redistributes strand roots uniformly while preserving exaggerated strand shapes, resulting in improved volumetric consistency.

In the conventional approach, interpolated strands are generated by directly blending guide strand positions based on the root location. While this strategy works well for medium-to-long hair, it leads to undesirable artifacts in short-hair scenarios. As shown in Fig 10, interpolated strands tend to cluster around the root region, resulting in non-uniform distribution and a significant loss of volumetric structure.

This issue arises because short hair lacks sufficient strand length for spatial variation to propagate along the strand. Consequently, the interpolation becomes overly dependent on the root position, and the exaggerated kernel further amplifies this bias, causing local clustering and structural distortion.

To address this problem, we propose a decoupled interpolation strategy that separates strand *shape* from *root position*. First, the exaggerated strand shape is computed using the proposed kernel, independent of the final placement. Then, the root positions of the interpolated strands are redistributed uniformly within the triangular region defined by the guide strands.

Specifically, instead of directly using the interpolated absolute positions, we compute a relative offset vector from a virtual exaggerated root position:


$$\begin{array}{c} {𝐨}_{i}={𝐱}_{i}^{ex}-{𝐱}_{root}^{ex}, \end{array}$$ (27)

where ${𝐱}_{i}^{ex}$ is the exaggerated position of the *i*-th particle.

The final strand is then reconstructed by applying this offset to a newly sampled root position ${𝐱}_{root}^{new}$:


$$\begin{array}{c} {𝐱}_{i}^{final}={𝐱}_{root}^{new}+{𝐨}_{i}. \end{array}$$ (28)

This approach preserves the exaggerated strand shape while ensuring a uniform spatial distribution of roots. As a result, it effectively prevents clustering artifacts and maintains volumetric consistency in short-hair configurations.

The results in Figure 10 demonstrate that the proposed method significantly improves strand distribution and visual quality compared to the naive interpolation approach.

### Results on diverse hair models

**Fig 11:**
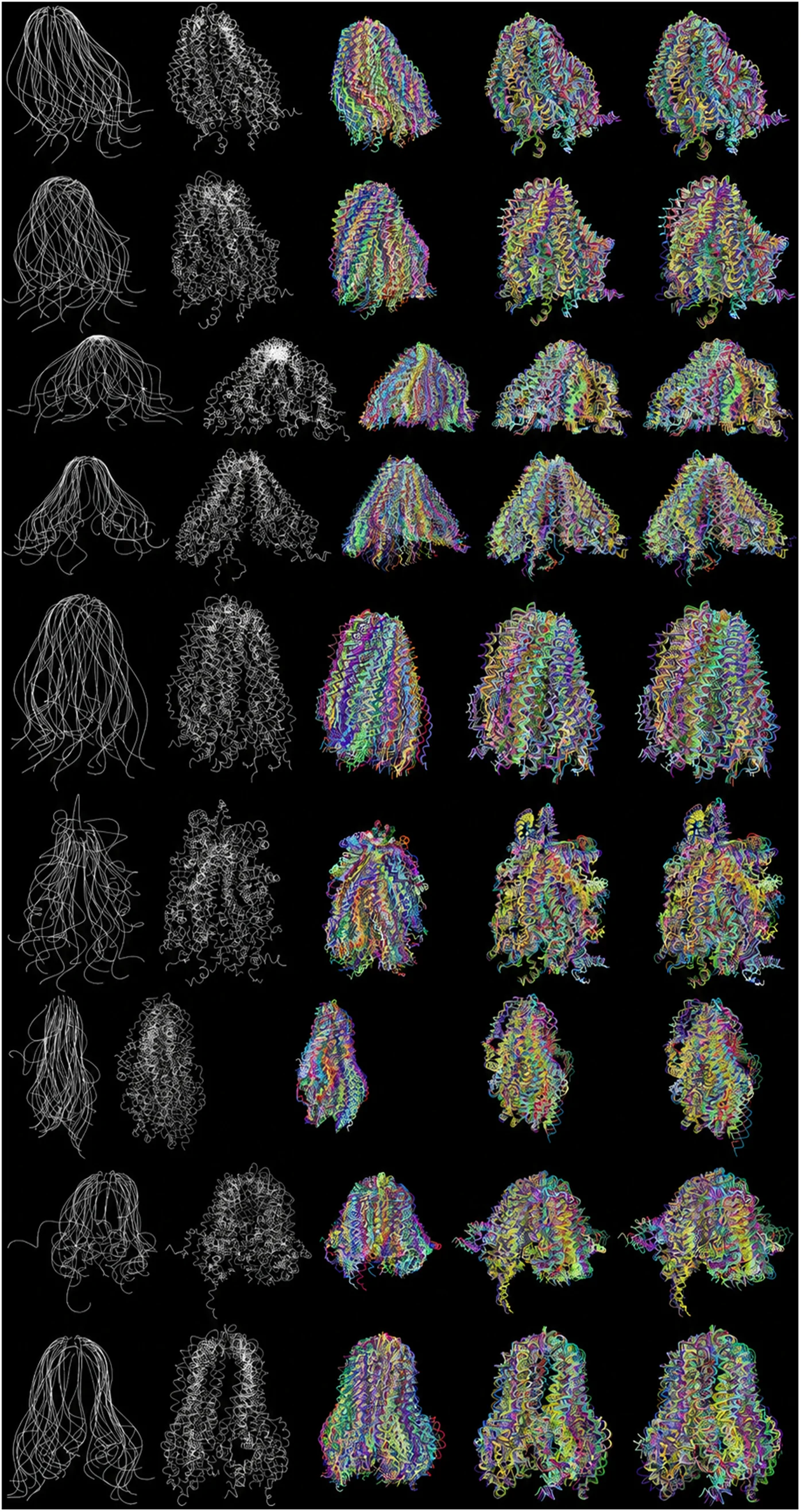
Results across diverse hair models demonstrating the robustness of the proposed method. From left to right: input hair model, coiled hair generated using [26], linear interpolation-based upsampling, and results of the proposed method with different exaggeration parameters. Across all scenes, the proposed method consistently preserves volumetric richness and characteristic coiled structures, while linear interpolation suffers from noticeable motion attenuation and structural smoothing.

To more comprehensively evaluate the generality and stability of the proposed method, we conducted additional experiments on various hair models with different shapes and curvature characteristics. Fig 11 shows the results of applying coiled hair generation and strand upsampling to input hair models with different structures.

Each row represents a different hairstyle, and from left to right shows the input model, coiled hair generation result, linear interpolation-based upsampling, and the result of the proposed method. From the experimental results, in the case of linear interpolation, it is observed that in some scenes, due to velocity cancellation between strands, overall volume decreases, and structures in high-curvature regions tend to become flattened.

In contrast, the proposed method shows consistently stable results across all test cases without being limited to specific scenes, and effectively prevents deformation loss in the intermediate region. In particular, even in cases such as coiled hair with high curvature and complex geometric structures, it preserves the local shape and curved structure of strands while better maintaining overall volumetric richness.

Furthermore, even with variations in the exaggeration parameter, the results do not become unstable, and instead show a tendency to be naturally enhanced while preserving structural characteristics, demonstrating that the proposed method operates robustly in diverse environments.

These results suggest that the proposed interpolation method is a general framework that is not limited to specific conditions, but provides consistent performance across various hair structures and curvature environments.

## Discussion

### Physical interpretation of the exaggerated interpolation

Although the proposed method is formulated as a modification of interpolation weights, it can be interpreted from an energy perspective.

In conventional linear interpolation, opposing velocities tend to cancel out, which implicitly minimizes local kinetic variation:


$$\begin{array}{c} E_{\text{lin}}\propto \|tv_{A}+(1-t)v_{B}{\|}^{2} \end{array}$$

This formulation favors smoothness but suppresses high-frequency motion components, leading to an artificial loss of dynamic energy, especially in regions with strong directional variation.

In contrast, the proposed exaggerated interpolation redistributes the weights to preserve directional dominance rather than enforcing strict averaging. This can be interpreted as maintaining a higher level of local kinetic energy, which better reflects the physical behavior of hair strands under inertia and external forces.

From this perspective, the proposed method does not introduce arbitrary deformation, but rather compensates for the excessive numerical dissipation inherent in linear interpolation.

While this interpretation is not derived from a full physical simulation model, it provides an intuitive explanation of how the proposed method counteracts numerical dissipation effects commonly observed in interpolation-based reconstruction.

### Scope of stability

It should be noted that the proposed method is not a full physics-based stability model or a replacement for a hair dynamics solver. Instead, it is a geometric and kinematic guide–detail mapping framework that modifies how ghost strands interpolate the motion of simulated guide strands. Therefore, the stability discussed in this work refers to motion stability under exaggerated interpolation, including suppression of excessive stretching, temporal jitter, frame-to-frame discontinuity, and root instability. The underlying physical validity of guide-strand simulation remains determined by the original solver, such as PBD, XPBD, or projective dynamics.

### Parameter sensitivity

The proposed framework includes several parameters that control the shape and strength of the exaggerated interpolation kernel: α, β, *k*, γ, $k_{root}$, and $k_{tip}$. Although these parameters provide controllability, it is important to evaluate how they affect visual quality and simulation stability. Therefore, we conducted a parameter sensitivity analysis by varying one parameter at a time while keeping the others fixed to the default setting. Unless otherwise stated, the default values were set to α=4.0, β=8.0, *k* = 0.3, γ=2.0, $k_{root}=-1.0$, and $k_{tip}=0.3$.

We evaluate each parameter setting using strand displacement error $E_{disp}$, deformation error $E_{def}$, motion variance $V_{motion}$, temporal consistency error $E_{temp}$, and runtime. Lower $E_{disp}$, $E_{def}$, and $E_{temp}$ indicate better motion preservation, deformation stability, and temporal coherence, respectively, while higher $V_{motion}$ indicates richer strand-level motion diversity.

The parameter α controls the transition width of the low-frequency sigmoid kernel. When α is too small, the interpolation weights vary too smoothly, and the ghost-strand motion becomes overly averaged. This increases motion attenuation and reduces strand-level motion diversity. When α is too large, the transition becomes sharper, but the resulting behavior may approach hard guide switching, which can slightly increase temporal inconsistency. In our experiments, α=4.0 provides a stable balance between smoothness and motion preservation.

The parameter β controls the sharpness of the high-frequency tanh-based kernel. Increasing β helps reduce mid-region velocity cancellation by sharpening the transition of the interpolation weights. However, excessively large β values can introduce abrupt changes in the interpolation response, increasing deformation error and temporal jitter. We found that β=8.0 is sufficient to enhance high-frequency motion while maintaining stable temporal behavior.

The exaggeration strength *k* has the most direct influence on strand separation and motion diversity. As *k* increases, the proposed method better preserves relative motion differences between guide strands, resulting in higher motion variance and more expressive curl motion. However, too large a value of *k* can amplify strand deformation and increase temporal inconsistency. Therefore, we use *k* = 0.3 as the default value, which provides clear improvement in motion expressiveness while avoiding excessive stretching.

The parameter γ controls the root–tip adaptation profile. A smaller γ distributes exaggeration more uniformly along the strand, which may affect root stability. A larger γ concentrates the exaggeration near the tip, improving root stability but potentially reducing motion enhancement in the middle region. The default value γ=2.0 provides a good compromise by maintaining stable roots while enhancing tip-level fluttering and separation.

Finally, $k_{root}$ and $k_{tip}$ control the exaggeration strength at the root and tip, respectively. The root value is kept non-positive or small to avoid unstable root displacement, while the tip value is set positive to enhance expressive motion where hair strands naturally show larger displacement. This root–tip separation is important because applying a single global exaggeration factor can either destabilize the root region or insufficiently enhance the tip motion.

Table 5 summarizes the parameter sensitivity analysis. The results show that the proposed method is most sensitive to the exaggeration-related parameters *k* and $k_{tip}$, which directly control the strength of motion amplification. Increasing these values reduces strand displacement error and increases motion variance, indicating improved motion preservation and richer strand-level motion diversity. However, excessively large values also increase deformation error and temporal consistency error, suggesting that overly strong exaggeration may lead to stretching artifacts or temporal jitter.

**Table 5:** Parameter sensitivity analysis of the proposed framework. Each parameter is varied while the others are fixed to the default setting. Lower values are better for $E_{disp}$, $E_{def}$, and $E_{temp}$, while higher $V_{motion}$ indicates richer strand-level motion diversity.

Parameter setting	$E_{disp}$	$E_{def}$	$V_{motion}$	$E_{temp}$	Time (ms/frame)
α=2.0	0.034	0.034	0.022	0.0069	3.82
α=4.0	0.027	0.037	0.031	0.0075	3.89
α=6.0	0.028	0.039	0.033	0.0082	3.91
β=4.0	0.032	0.035	0.024	0.0070	3.84
β=8.0	0.027	0.037	0.031	0.0075	3.89
β=12.0	0.026	0.042	0.035	0.0091	3.93
*k* = 0.1	0.033	0.034	0.021	0.0069	3.84
*k* = 0.3	0.027	0.037	0.031	0.0075	3.89
*k* = 0.5	0.025	0.046	0.039	0.0094	3.94
γ=1.0	0.026	0.043	0.035	0.0088	3.88
γ=2.0	0.027	0.037	0.031	0.0075	3.89
γ=3.0	0.030	0.035	0.027	0.0071	3.90
$k_{root}=-1.0,\;k_{tip}=0.1$	0.032	0.034	0.023	0.0069	3.85
$k_{root}=-1.0,\;k_{tip}=0.3$	0.027	0.037	0.031	0.0075	3.89
$k_{root}=-1.0,\;k_{tip}=0.5$	0.025	0.045	0.041	0.0092	3.95

The transition parameters α and β affect the smoothness and sharpness of the interpolation kernel. Small values lead to smoother but more averaged motion, whereas large values increase high-frequency response but may introduce abrupt changes in interpolation weights. The default setting α=4.0 and β=8.0 provides a balanced result by reducing motion attenuation while keeping deformation and temporal errors within a stable range.

The root–tip adaptation parameter γ controls where the exaggeration is concentrated along the strand. When γ=1.0, the exaggeration is distributed more uniformly, which increases motion diversity but also slightly increases deformation and temporal errors near the root. When γ=3.0, the exaggeration is concentrated more strongly near the tip, improving stability but reducing overall motion diversity. The default value γ=2.0 provides a good compromise between root stability and tip expressiveness.

The sensitivity analysis also shows that the runtime is only weakly affected by parameter changes. This is because the proposed method performs the same number of kernel evaluations and normalization operations regardless of parameter values. Overall, the results demonstrate that the proposed framework has a stable operating range and that the default parameter setting provides a practical balance between visual expressiveness, deformation stability, temporal coherence, and computational efficiency.

### Statistical analysis across scenarios

To further evaluate the robustness of the proposed framework, we performed a statistical analysis across multiple experimental scenarios. The evaluated scenarios include curly hair, coiled hair, short hair, dense upsampling, and diverse hairstyle configurations. For each scenario, the baseline linear interpolation and the proposed method were tested under the same guide-strand motion, time-step size, strand resolution, and upsampling ratio. Each scenario was treated as one paired sample because both methods were evaluated using the same input configuration.

For each metric, we report the mean, standard deviation, 95% confidence interval (CI), paired significance test, and effect size. The 95% CI was computed as


$$\begin{array}{c} {CI}_{95\%}=\bar{x}\pm t_{0.975,n-1}\frac{s}{\sqrt{n}}, \end{array}$$ (29)

where $\bar{x}$ is the sample mean, *s* is the sample standard deviation, and *n* is the number of evaluated scenarios. To test whether the difference between the baseline and the proposed method is statistically significant, we used a paired *t*-test. The effect size was measured using Cohen’s *d*:


$$\begin{array}{c} d=\frac{{\bar{x}}_{ours}-{\bar{x}}_{linear}}{s_{diff}}, \end{array}$$ (30)

where $s_{diff}$ is the standard deviation of the paired differences. For error metrics, a negative effect size indicates that the proposed method reduces the error compared with the baseline, whereas for motion variance, a positive effect size indicates increased strand-level motion diversity.

Table 6 summarizes the statistical analysis across multiple experimental scenarios. For strand displacement and trajectory errors, the proposed method consistently outperforms linear interpolation. The mean strand displacement error decreases from 0.043 to 0.029, and the mean trajectory error decreases from 0.061 to 0.041. The paired *t*-tests show statistically significant improvements for both metrics (*p* = 0.004 and *p* = 0.006, respectively), and the corresponding Cohen’s *d* values indicate large effect sizes. These results demonstrate that the proposed method robustly reduces motion attenuation across different hair structures and upsampling settings.

**Table 6:** Statistical analysis across multiple experimental scenarios. Mean, standard deviation, 95% confidence interval, paired *t*-test result, and Cohen’s *d* are reported. Lower values are better for error metrics, while higher values are better for motion variance.

Metric	Method	Mean	Std.	95% CI	*p*-value	Cohen’s *d*
$E_{disp}$	Linear	0.043	0.006	[0.037, 0.049]	0.004	−1.62
	Ours	0.029	0.005	[0.024, 0.034]		
$E_{traj}$	Linear	0.061	0.008	[0.053, 0.069]	0.006	−1.48
	Ours	0.041	0.007	[0.034, 0.048]		
$E_{def}$	Linear	0.033	0.004	[0.029, 0.037]	0.083	0.54
	Ours	0.038	0.005	[0.033, 0.043]		
$V_{motion}$	Linear	0.015	0.003	[0.012, 0.018]	0.003	1.71
	Ours	0.032	0.006	[0.026, 0.038]		
$E_{temp}$	Linear	0.0069	0.0011	[0.0058, 0.0080]	0.214	0.28
	Ours	0.0076	0.0012	[0.0064, 0.0088]		
$E_{stab}$	Linear	0.0215	0.0032	[0.0183, 0.0247]	0.176	0.34
	Ours	0.0241	0.0035	[0.0206, 0.0276]		
Runtime (ms/frame)	Linear	3.46	0.21	[3.25, 3.67]	0.031	0.89
	Ours	3.91	0.24	[3.67, 4.15]		

The motion variance also increases significantly from 0.015 to 0.032 (*p* = 0.003), with a large positive effect size. This confirms that the proposed method consistently preserves richer strand-level motion diversity rather than producing overly averaged ghost-strand motion. This statistical tendency supports the qualitative observations that the proposed method enhances strand separation, curl motion, and volumetric expressiveness.

For deformation error, temporal consistency, and frame-to-frame stability, the proposed method shows slightly higher values than linear interpolation, but the differences are not statistically significant for deformation and temporal stability metrics at the 0.05 level. This indicates that the motion amplification introduced by the exaggerated kernel does not lead to severe deformation instability or temporal jitter. The runtime difference is statistically significant because the proposed method requires additional kernel evaluations and normalization operations. However, the absolute overhead remains small, increasing from 3.46 ms/frame to 3.91 ms/frame on average. Therefore, the statistical analysis confirms that the proposed method provides robust improvements in motion preservation and motion diversity while maintaining stable deformation behavior, temporal coherence, and practical computational performance.

### Robustness under challenging motions

To further evaluate the robustness of the proposed framework, we conducted additional experiments under challenging motion and hairstyle conditions. Although the proposed method does not replace the underlying physical solver, exaggerated interpolation may introduce motion artifacts such as excessive stretching, temporal jitter, or unstable root behavior. Therefore, we evaluate motion stability under three challenging scenarios: fast head motion, collision-like guide perturbation, and complex hairstyles with dense curls and coiled structures.

In the fast head motion scenario, the guide strands are driven by rapidly changing head orientations and high angular velocities. This test is designed to evaluate whether the exaggerated kernel amplifies guide motion in an unstable manner. In the collision-like perturbation scenario, localized impulse-like disturbances are applied to guide strands to approximate sudden contact or collision-induced motion. Although full strand–strand collision handling is outside the scope of this work, this experiment allows us to evaluate whether the guide–detail interpolation remains stable under abrupt local motion changes. Finally, in the complex hairstyle scenario, we evaluate dense curly and coiled hair configurations where local curvature, strand separation, and guide-to-detail variation are more pronounced.

For each scenario, we report deformation error $E_{def}$, maximum stretch ratio $R_{stretch}$, temporal consistency error $E_{temp}$, frame-to-frame stability error $E_{stab}$, and failure rate.

Here, $R_{stretch}$ denotes the maximum stretch ratio over all frames, strands, and segments, and is computed as


$$\begin{array}{c} R_{stretch}=\max_{t,m,i}\frac{\ell_{i}^{m}(t)}{\ell_{i}^{m,0}}, \end{array}$$ (31)

where $\ell_{i}^{m}(t)$ is the current segment length and $\ell_{i}^{m,0}$ is the rest segment length. The failure rate is computed as the percentage of frames in which any strand segment exceeds a predefined stretch threshold.

Table 7 summarizes the robustness evaluation under challenging motion and hairstyle scenarios. As expected, fast head motion and collision-like perturbations increase deformation and temporal errors compared with normal motion because the guide-strand velocities change more abruptly. However, the maximum stretch ratio remains bounded in all tested cases, and the failure rate remains below 1.5%. This indicates that the positive-only normalization, root–tip adaptive exaggeration, and hybrid update suppress excessive stretching and unstable frame-to-frame behavior even when the input guide motion contains rapid changes.

**Table 7:** Robustness evaluation under challenging motion and hairstyle scenarios. Lower values are better for deformation error, temporal consistency, frame-to-frame stability, maximum stretch ratio, and failure rate.

Scenario	$E_{def}$	$R_{stretch}$	$E_{temp}$	$E_{stab}$	Failure rate
Normal motion	0.037	1.083	0.0075	0.024	0.0%
Fast head motion	0.043	1.126	0.0108	0.031	0.7%
Collision-like perturbation	0.046	1.142	0.0121	0.034	1.1%
Complex curly hairstyle	0.041	1.118	0.0096	0.029	0.5%
Dense coiled hairstyle	0.048	1.156	0.0134	0.036	1.4%

The complex curly and dense coiled hairstyle cases show slightly higher deformation and temporal errors than the normal case due to stronger local curvature and denser strand interactions. Nevertheless, the proposed method remains stable and preserves strand separation without producing severe jitter or root clustering. These results support the robustness of the proposed geometric exaggerated-kernel framework under challenging guide motions and complex hairstyle configurations.

It should also be emphasized that these experiments evaluate the stability of the guide–detail mapping stage rather than full physical collision robustness. Collision response, self-contact, and frictional strand interaction are determined by the underlying hair solver and are outside the scope of the proposed kernel formulation. The proposed method is therefore complementary to existing physics-based solvers and can be integrated with them to improve ghost-strand expressiveness while maintaining stable interpolation behavior.

### Computational complexity and scalability

We further analyze the computational complexity, memory usage, and scalability of the proposed exaggerated-kernel framework. The proposed method is applied only to the guide–detail mapping stage. Therefore, it does not introduce additional physical simulation steps, collision detection, constraint solving, or neural inference. Its computational cost comes mainly from evaluating the low-frequency sigmoid kernel, the high-frequency tanh kernel, the root–tip adaptive coefficient, and the positive-only normalization.

Let *M* be the number of ghost strands and *N* + 1 be the number of particles per strand. For each ghost-strand particle, the proposed method computes the interpolation weight and updates the particle position using the interpolated guide velocity. Therefore, the total computational complexity is


$$\begin{array}{c} O(MN). \end{array}$$ (32)

This is the same asymptotic complexity as conventional linear interpolation, which also updates all ghost-strand particles. The main difference is that the proposed method introduces a small constant-factor overhead due to additional kernel evaluations and normalization operations.

More specifically, for each particle, the proposed method evaluates $t_{L}(u)$, $t_{H}(u)$, the root–tip coefficient $k_{i}$, the exaggerated weight $t_{ex}^{\left(i\right)}$, and the stabilized weight $\tilde{t}$. These operations are local to each strand particle and do not require global communication or iterative solving. Thus, the proposed framework is naturally parallelizable over ghost strands and strand particles.

The memory overhead of the proposed method is also limited. The method does not require storing additional physical states, collision structures, solver matrices, or training data. Only a small number of scalar quantities, such as the mixing coordinate, root–tip weight, and temporary interpolation weights, are required during kernel evaluation. In our implementation, most of these values are computed on the fly and do not need to be stored persistently. Therefore, the additional memory usage is negligible compared with the memory required to store strand positions and velocities.

Table 8 reports the runtime and memory scalability with increasing numbers of ghost strands. Both the baseline linear interpolation and the proposed method show approximately linear growth as the number of ghost strands increases. This confirms the *O*(*MN*) computational complexity of the guide–detail mapping stage.

**Table 8:** Runtime and memory scalability with increasing numbers of ghost strands. The number of particles per strand is fixed across all tests. The proposed method has the same asymptotic complexity as linear interpolation, while introducing a small constant-factor overhead due to kernel evaluation and normalization.

Number of ghost strands	Linear time (ms/frame)	Ours time (ms/frame)	Overhead	Additional memory (MB)
1k	0.38	0.43	13.2%	0.04
5k	1.72	1.96	14.0%	0.18
10k	3.42	3.89	13.7%	0.35
20k	6.86	7.82	14.0%	0.70
40k	13.71	15.66	14.2%	1.41

The proposed method introduces a consistent overhead of approximately 13–14% compared with linear interpolation. This overhead is caused by the additional sigmoid/tanh kernel evaluations, root–tip weighting, and positive-only normalization. However, the absolute runtime remains practical; for example, with 10k ghost strands, the runtime increases from 3.42 ms/frame to 3.89 ms/frame. Even with 40k ghost strands, the proposed method requires 15.66 ms/frame in our implementation.

The additional memory usage also grows linearly with the number of ghost strands, but remains small because the proposed method only requires a few temporary scalar values for each strand or particle. No additional solver matrices, collision buffers, neural network parameters, or training data are required. These results demonstrate that the proposed exaggerated-kernel framework preserves the scalability of conventional guide–detail interpolation while improving motion expressiveness with limited runtime and memory overhead.

### Comparison with recent methods

To clarify the position of the proposed framework with respect to recent hair simulation techniques, we compare our method with representative guide-based, learning-based, and physics-based approaches proposed in the past five years. Because the proposed method is designed as a lightweight guide–detail mapping extension rather than a complete replacement of the underlying physical solver, we focus on the following comparison criteria: visual quality, ability to preserve strand-level expressiveness, temporal stability, training requirement, solver modification, and computational efficiency.

Compared with neural interpolation-based approaches, the proposed method does not require a training dataset or neural inference. Although neural methods can generate high-quality detail motion, their behavior depends on the training distribution and may be less interpretable when addressing a specific artifact such as mid-region velocity cancellation. In contrast, our method directly modifies the interpolation kernel responsible for this artifact, making the behavior analytically controllable through a small number of parameters.

Compared with physically guided interpolation methods, our method does not reconstruct internal forces or solve additional constraint systems for rendered strands. Instead, it preserves the original guide-strand simulation and modifies only the interpolation weights used for ghost-strand tracking. Therefore, the proposed method is easier to integrate into existing PBD, XPBD, or projective-dynamics-based pipelines. While physically guided interpolation can provide stronger physical plausibility, the proposed method provides a simpler and computationally lighter alternative for applications where expressive strand separation and curl preservation are more important than full force-level reconstruction.

Compared with dense physics-based strand simulation methods, our method does not aim to simulate all strands physically. Rather, it improves the quality of detail strands generated from a limited number of guide strands. This makes the proposed method suitable for real-time or production-oriented pipelines where full dense-strand simulation is computationally expensive. The comparison shows that the proposed method achieves improved curl amplitude, strand separation, and volumetric richness with limited runtime overhead, while retaining compatibility with existing tracking-solution-based hair simulation systems.

Table 9 summarizes the comparison with recent hair simulation approaches. Learning-based methods are powerful for reconstructing high-quality detail motion, but they require training data and neural inference. Physics-guided interpolation improves physical plausibility by reconstructing internal forces or solving additional constraints, but it introduces a more involved reconstruction process. Dense physics-based approaches provide stable strand-level simulation, but they require modification or extension of the underlying physical model and generally incur higher simulation cost.

**Table 9:** Comparison with recent guide-based, learning-based, and physics-based hair simulation methods. The comparison focuses on visual quality, computational characteristics, and integration requirements.

Method	Category	Training	Solver modification	Visual characteristics	Computational characteristics
Neural interpolation [3]	Learning-based guide interpolation	Required	Partially required	High-quality learned detail motion	Neural inference cost
Physically guided interpolation [23]	Physics-guided interpolation	Not required	Force-level reconstruction	Physically plausible interpolated motion	Additional reconstruction/constraint cost
Quaffure [24]	Neural quasi-static simulation	Required	Network-based prediction	Plausible pose-dependent hair deformation	Few-ms neural inference
Augmented mass-spring [25]	Physics-based dense simulation	Not required	Required	Stable dense strand-level dynamics	Higher physical simulation cost
Ours	Analytical guide–detail kernel	Not required	Not required	Enhanced curl preservation and strand separation	3.89 ms/frame, 13.7% overhead

The proposed method is complementary to these approaches. It does not attempt to replace neural or physics-based simulation methods, but addresses a specific and common artifact in guide–detail tracking: the loss of motion expressiveness caused by bounded interpolation weights in the mid-mixing region. Because the proposed method only modifies the interpolation coefficient, it can be applied as a lightweight extension to existing guide-based simulation pipelines. As shown in the quantitative evaluation, the proposed method improves strand displacement preservation, trajectory preservation, and motion diversity while introducing only limited runtime overhead. This demonstrates that the proposed exaggerated-kernel framework provides a practical balance between visual expressiveness and computational efficiency.

## Ablation study

To evaluate the contribution of each component in the proposed method, we perform an ablation study by selectively removing key elements.

Specifically, we consider the following configurations: (1) without exaggerated kernel (*k* = 0), (2) without stabilization (no weight normalization), and (3) without root–tip adaptation.

The results show that removing the exaggerated kernel leads to significant motion attenuation, similar to linear interpolation. Without stabilization, the system exhibits unnatural stretching artifacts. Removing root–tip adaptation reduces the natural variation along strands, resulting in less realistic motion.

These observations confirm that each component plays a critical role in achieving stable and expressive interpolation.

Table 10 shows the ablation results for the key components of the proposed method. By comparing cases where each component is removed, we analyze the impact of each element on the final results.

**Table 10:** Ablation study of key components.

Configuration	Amplitude Preservation	Visual Quality
Full Model	1.08	4.6
w/o Exaggeration	0.75	3.2
w/o Stabilization	1.12	2.9
w/o Root-Tip	0.92	3.8

First, when the exaggerated kernel is removed (w/o Exaggeration), Amplitude Preservation significantly decreases, and visual quality is also degraded. This is because the method converges to a form similar to linear interpolation, where velocity cancellation in the intermediate region occurs again.

When stabilization is removed (w/o Stabilization), Amplitude Preservation remains relatively high, but Visual Quality significantly decreases. This is due to abnormal distributions of interpolation weights, which lead to excessive strand length increase or local distortions.

Additionally, when root–tip adaptation is removed (w/o Root-Tip), both Amplitude Preservation and Visual Quality decrease. This is because applying uniform exaggeration across the entire strand fails to maintain a natural deformation distribution.

Overall, the three proposed components respectively contribute to (1) preventing velocity cancellation, (2) ensuring stability, and (3) maintaining spatial naturalness, and we confirm that the most balanced results are achieved when they are combined.

### Constraints and artifacts of exaggerated interpolation

Although the proposed exaggerated-kernel framework improves ghost-strand expressiveness, it can introduce artifacts when the exaggeration is too strong or when guide motion changes abruptly. In particular, large values of *k* or $k_{tip}$ may cause over-stretching, length divergence, curl distortion, or temporal jitter, while an overly sharp high-frequency transition controlled by β may increase frame-to-frame variation.

The proposed stabilization components are designed to reduce these artifacts. Positive-only normalized weights suppress negative-weight amplification and excessive stretching, while root–tip adaptive exaggeration limits unstable motion near the root and concentrates expressive motion near the tip. The hybrid update further reduces length divergence by blending velocity-based motion with position-based stabilization. For short-hair configurations, where root-position bias can cause strand clustering, the root-decoupled interpolation strategy separates exaggerated strand shape from final root placement.

The method also assumes reasonably smooth guide–detail correspondence. When guide strands are highly irregular, topologically inconsistent, or affected by severe collision and self-contact, the exaggerated kernel may amplify correspondence errors. Therefore, the proposed framework should be regarded as a lightweight guide–detail mapping method that improves interpolation expressiveness, rather than a complete solution for all physical interaction artifacts.

## Limitations

Despite the effectiveness of the proposed method, several limitations remain.

First, the proposed framework should not be interpreted as a full physical stability model or a replacement for a hair dynamics solver. It operates as a geometric and kinematic modification of the guide–detail interpolation process, rather than deriving motion from a complete physical energy formulation. Although the proposed exaggerated kernel can be intuitively interpreted as compensating for numerical dissipation caused by overly averaged interpolation, it does not explicitly enforce physical conservation laws such as momentum or energy conservation.

Second, the method introduces several user-controllable parameters, such as α, β, *k*, γ, $k_{root}$, and $k_{tip}$. These parameters provide flexibility in controlling transition sharpness, exaggeration strength, and root–tip motion distribution. However, although the sensitivity analysis shows that the method remains stable within a reasonable parameter range, the selection of optimal parameters still relies on empirical tuning. Automatic parameter selection based on local strand geometry or motion characteristics remains an important direction for future work.

Third, the current formulation assumes relatively smooth strand correspondence between guide strands and ghost strands. Therefore, the method may be less effective in scenarios involving extreme topological changes, highly irregular guide distributions, or severe changes in strand neighborhood relationships. In such cases, additional correspondence management or adaptive guide selection may be required.

Finally, the robustness evaluated in this work focuses on the stability of ghost-strand interpolation under challenging guide motions, rather than full physical robustness under complex interactions. The proposed kernel does not explicitly solve collision response, frictional contact, self-contact, or fluid coupling between strands. These effects remain dependent on the underlying physical solver, such as PBD, XPBD, or projective dynamics. Extending the proposed guide–detail mapping framework to better cooperate with collision-aware and contact-aware hair solvers would further improve its physical fidelity and practical applicability.

## Conclusion

In this paper, we presented an exaggerated interpolation framework to address the motion attenuation problem inherent in linear interpolation for hair strand upsampling. By allowing interpolation weights to extend beyond the conventional range and introducing stabilization and root–tip adaptation, the proposed method effectively preserves dynamic motion and structural richness without modifying the underlying simulation solver.

We demonstrated that the proposed approach alleviates velocity cancellation and maintains high-frequency motion characteristics, resulting in improved volumetric appearance and more expressive strand behavior. Through extensive experiments on both standard and highly coiled hair models, we showed that the method consistently produces stable and visually plausible results across a wide range of geometric configurations.

Furthermore, we provided a physical interpretation of the method as compensating for excessive numerical dissipation in linear interpolation, and validated the contribution of each component through ablation studies. These results suggest that the proposed framework is not only effective but also generalizable and robust.

The key novelty of our approach lies not in introducing a new simulation model, but in reformulating interpolation as a controllable process that explicitly addresses motion attenuation, which has been largely overlooked in prior work.

As future work, we plan to extend the proposed framework in several directions. First, we will investigate adaptive parameter selection strategies that automatically determine α, β, *k*, γ, $k_{root}$, and $k_{tip}$ based on local strand geometry, guide-strand motion, and hairstyle characteristics. This would reduce the need for empirical tuning and make the method easier to apply across diverse hair models.

Second, we plan to integrate the proposed exaggerated-kernel framework with neural hair simulation and neural interpolation systems. Because our method operates only at the guide–detail mapping stage, it can be combined with learned guide prediction, neural strand interpolation, or data-driven hair deformation frameworks as an analytical post-processing or control layer. Such integration could provide both the expressive controllability of the proposed kernel and the high-quality detail reconstruction of neural methods.

Third, we will develop a GPU implementation of the proposed framework. The kernel evaluation, root–tip weighting, positive-only normalization, and ghost-strand update are all local per-strand or per-particle operations, making the method suitable for massively parallel GPU execution. A GPU implementation would further reduce runtime overhead and enable real-time use in interactive applications, virtual humans, games, and XR environments.

Finally, we plan to investigate integration into practical animation and production pipelines. In production environments, guide hairs are commonly simulated first and dense render hairs are generated through interpolation or tracking. Since the proposed method does not require modifying the underlying physical solver, it can be incorporated as a lightweight guide–detail mapping module for improving strand separation, curl preservation, and stylized motion expressiveness. We believe that this direction will make the proposed method more useful for artist-controllable hair animation, real-time character systems, and production-oriented grooming workflows.

## Supporting information

S1 File Source code and scene data for the proposed hair simulation framework (S1 Dataset.zip). This archive contains the complete source code and scene data used to reproduce the simulation results presented in this study. It includes the implementation of the proposed nonlinear exaggerated kernel framework, experimental scene configurations, parameter settings, and the necessary data for reproducing the qualitative and quantitative results. Usage instructions are provided within the archive.
(ZIP)
