# Supplementary material for: A nonlinear exaggerated kernel framework for expressive hair motion in tracking solution-based simulation
Source: PLoS One. 2026 Aug 31;21(8):e0356631. doi: 10.1371/journal.pone.0356631 (PMC13529024; doi:10.1371/journal.pone.0356631)
Supplement: Supplementary file 1 [file pone.0356631.s003.docx]

**Reviewer#1, Concern#1:** The experimental validation is mainly qualitative. While the comparisons in the visual sense show increased expressiveness, there is a lack of quantitative evaluation in the manuscript. Other numerical measurements like: Strand Displacement Error, Trajectory Error, Deformation Error, Motion Variance, Temporal Consistency, Frame-to-Frame Stability, Computational Overhead and Runtime analysis should be reported.

**Author response:** We thank the reviewer for this important comment. We agree that the original manuscript relied mainly on qualitative visual comparisons and amplitude-based analysis, which was not sufficient to fully validate the proposed framework from a numerical perspective. In response to this concern, we have substantially revised the Experimental Results section by adding a new quantitative evaluation.

Specifically, we added numerical measurements for Strand Displacement Error, Trajectory Error, Deformation Error, Motion Variance, Temporal Consistency, Frame-to-Frame Stability, and Runtime. These metrics were computed under the same guide-strand motion, time-step size, strand resolution, and upsampling ratio for both the baseline linear interpolation and the proposed exaggerated-kernel interpolation. Through this evaluation, we aimed to verify not only whether the proposed method visually enhances strand motion, but also whether it preserves guide-driven motion, maintains stable deformation, avoids temporal jitter, and remains computationally practical.

The added results show that the proposed method reduces Strand Displacement Error from 0.041 to 0.027 and Trajectory Error from 0.058 to 0.039. These results indicate that the proposed exaggerated-kernel interpolation better preserves the intended ghost-strand motion and alleviates the motion attenuation caused by conventional neutral blending in the mid-mixing region. In addition, Motion Variance increases from 0.014 to 0.031, demonstrating that the proposed method preserves richer strand-level motion diversity rather than collapsing ghost strands into overly averaged motion.

We also evaluated deformation and temporal stability. The Deformation Error slightly increases from 0.032 to 0.037, which is expected because the proposed method intentionally amplifies relative motion differences between guide strands to enhance strand separation and curl expressiveness. However, this increase remains bounded due to the proposed positive-only normalization and root–tip adaptive weighting. Moreover, the Temporal Consistency Error and Frame-to-Frame Stability Error remain comparable to the baseline, increasing only from 0.0068 to 0.0075 and from 0.021 to 0.024, respectively. These results confirm that the proposed exaggerated kernel does not introduce severe temporal jitter or unstable frame-wise motion.

Finally, we added runtime analysis to evaluate computational overhead. The runtime increases from 3.42 ms/frame for linear interpolation to 3.89 ms/frame for the proposed method, corresponding to an overhead of approximately 13.7%. Since the proposed framework only modifies the interpolation weight computation in the guide–detail mapping stage and does not require additional physical simulation, collision handling, or solver iterations, the additional cost remains limited.

Accordingly, the revised manuscript now includes a dedicated quantitative evaluation table and accompanying analysis. This revision clarifies that the proposed method improves not only visual expressiveness, but also strand-level motion preservation, motion diversity, temporal coherence, and practical runtime efficiency.

**Revision:** We added a new subsection, **Quantitative Evaluation**, in the Experimental Results section. The new subsection defines the evaluation metrics, reports the quantitative comparison between linear interpolation and the proposed method, and discusses the computational overhead and temporal stability of the proposed framework. (see page 18 in revised manuscript: **"Quantitative Evaluation…"**)

**Reviewer#1, Concern#2:** No statistics have been provided. More than one experimental scenario will be evaluated, so both appropriate statistics (mean, standard deviation, confidence intervals, significance tests) and effect sizes (where applicable) should be provided to demonstrate the robustness of the proposed approach.

**Author response:** We thank the reviewer for this valuable comment. We agree that reporting only representative numerical values is not sufficient to demonstrate the robustness of the proposed approach across multiple experimental scenarios. In the original manuscript, the evaluation mainly focused on qualitative comparisons and representative quantitative values, but it did not provide sufficient statistical evidence such as standard deviations, confidence intervals, significance tests, or effect sizes.

To address this issue, we added a new statistical analysis subsection in the revised manuscript. In this subsection, we evaluate the proposed method across multiple experimental scenarios, including curly hair, coiled hair, short hair, dense upsampling, and diverse hairstyle configurations. For each scenario, the baseline linear interpolation and the proposed method were tested under the same guide-strand motion, time-step size, strand resolution, and upsampling ratio. Since both methods were evaluated on the same input configuration, each scenario was treated as a paired sample.

In the revised manuscript, we now report the mean, standard deviation, 95% confidence interval, paired significance test, and effect size for each quantitative metric. Specifically, we used paired t-tests to evaluate whether the difference between the baseline and the proposed method is statistically significant. We also reported Cohen’s d to quantify the effect size. The evaluated metrics include Strand Displacement Error, Trajectory Error, Deformation Error, Motion Variance, Temporal Consistency, Frame-to-Frame Stability, and Runtime.

The added statistical results show that the proposed method consistently improves the main motion-preservation metrics. The mean Strand Displacement Error decreases from 0.043 to 0.029, and the mean Trajectory Error decreases from 0.061 to 0.041. These improvements are statistically significant, with p-values of 0.004 and 0.006, respectively. The corresponding Cohen’s d values also indicate large effect sizes, demonstrating that the proposed method robustly reduces motion attenuation across different hair structures and upsampling conditions.

The Motion Variance also increases significantly from 0.015 to 0.032, with a p-value of 0.003 and a large positive effect size. This result supports the main purpose of the proposed exaggerated-kernel framework: instead of collapsing ghost strands into overly averaged motion, the proposed method preserves richer strand-level motion diversity. This statistical tendency is consistent with the qualitative observations that the proposed method enhances strand separation, curl motion, and volumetric expressiveness.

For Deformation Error, Temporal Consistency, and Frame-to-Frame Stability, the proposed method shows slightly higher values than the baseline because it intentionally amplifies relative motion differences between guide strands. However, these increases remain small, and the differences in deformation and temporal stability metrics are not statistically significant at the 0.05 level. This indicates that the proposed method improves motion expressiveness without introducing severe deformation instability or temporal jitter. Runtime increases moderately due to additional kernel evaluations and normalization operations, but the absolute overhead remains limited.

Accordingly, the revised manuscript now includes statistical evidence showing that the proposed framework is not effective only in selected visual examples, but provides robust improvements across multiple experimental scenarios. We believe this revision directly addresses the reviewer’s concern by providing mean and standard deviation values, confidence intervals, significance tests, and effect sizes.

**Revision:** We added a new subsection, **Statistical Analysis across Scenarios**, in the Experimental Results section. The new subsection reports the mean, standard deviation, 95% confidence interval, paired t-test results, and Cohen’s d for each evaluation metric across multiple experimental scenarios. We also added a statistical analysis table and accompanying discussion to demonstrate the robustness of the proposed approach. (see page 26 in revised manuscript: **"Statistical Analysis across …"**)

**Reviewer#1, Concern#3:** Limited comparison with current and modern techniques. The manuscript should provide comparisons to recent (past 5 years) hair simulation methods based on guides, learning and physics. Comparisons of performance should include both visual quality and computational efficiency.

**Author response:** We thank the reviewer for this important suggestion. We agree that the original manuscript did not sufficiently compare the proposed framework with recent hair simulation techniques, especially methods proposed in the past five years. Although the original manuscript discussed guide-based tracking, neural interpolation, and physics-based simulation in the Related Work section, the comparison with modern guide-based, learning-based, and physics-based approaches was not sufficiently explicit in terms of visual quality and computational efficiency.

To address this concern, we revised the manuscript by adding a new subsection, **Comparison with Recent Methods**. In this subsection, we compare the proposed framework with representative recent hair simulation approaches, including learning-based guide interpolation, physically guided hair interpolation, neural quasi-static hair deformation, and dense physics-based strand simulation. We also added recent references to better position the proposed method within the current research landscape.

The revised comparison clarifies that recent learning-based methods can generate high-quality detail motion, but they typically require training datasets and neural inference. Their behavior can also depend on the training distribution, which may make it less straightforward to directly control a specific interpolation artifact such as mid-region velocity cancellation. In contrast, the proposed method directly modifies the interpolation kernel responsible for this artifact, making the behavior analytically controllable through a small number of parameters.

We also compare the proposed method with recent physically guided interpolation methods. Such methods improve physical plausibility by interpolating internal forces or reconstructing rendered hair motion through additional physical constraints. However, they require a more involved reconstruction or constraint-solving process. By contrast, the proposed method does not reconstruct internal forces or introduce additional solver iterations. It preserves the original guide-strand simulation and modifies only the interpolation weights used in the guide–detail mapping stage. Therefore, it can be more easily integrated into existing PBD, XPBD, or projective-dynamics-based pipelines.

In addition, we compare the proposed framework with dense physics-based strand simulation methods. Dense physics-based methods can provide stable strand-level dynamics, but they generally require modification or extension of the underlying simulation model and involve higher computational cost. The proposed method does not aim to replace full dense-strand simulation. Instead, it improves the quality of detail strands generated from a limited number of guide strands, making it suitable for real-time or production-oriented tracking-solution-based pipelines.

We further added a comparison table summarizing visual characteristics, training requirements, solver modification requirements, and computational characteristics. The added comparison shows that the proposed method improves curl preservation, strand separation, and volumetric richness while requiring no training, no neural inference, and no solver modification. In terms of computational efficiency, the proposed method increases runtime only from 3.42 ms/frame to 3.89 ms/frame, corresponding to approximately 13.7% overhead. This limited overhead mainly comes from sigmoid/tanh kernel evaluation, root–tip weighting, and positive-only normalization.

Accordingly, the revised manuscript now provides a clearer comparison with current guide-based, learning-based, and physics-based hair simulation methods. This revision clarifies that the proposed method is not intended to replace neural or full physics-based simulation methods, but serves as a lightweight and analytically controllable extension for improving motion expressiveness in guide–detail hair simulation pipelines.

**Revision:** We added a new subsection, **Comparison with Recent Methods**, to the Discussion section. We also added recent references and a comparison table covering visual quality, computational efficiency, training requirement, and solver modification. This revision better positions the proposed method with respect to modern guide-based, learning-based, and physics-based hair simulation techniques. (see page 30 in revised manuscript: **"Comparison with Recent Methods …"**)

**Reviewer#1, Concern#4:** The reproducibility of the work should be improved. The manuscript indicates that source code and datasets will be made available following acceptance, but PLOS ONE demands that the underlying data which supports the findings must be made available during the review process or in a public repository. A repository link and/or a reviewer access should be offered.

**Author response:** To improve reproducibility, we will provide the full source code through the following GitHub repository: <https://github.com/dohee02/HairSimulation-ExaggerationKernel.git> (see page 34 in revised manuscript: **"Data and Code Availability …"**)

**Reviewer#1, Concern#5:** The proposed method has several hyper-parameters (α, β, k, γ, kroot, ktip) but sensitivity analysis is not provided. A systematic evaluation of these parameters on the quality and stability of the simulations should be done.

**Author response:** We thank the reviewer for this important comment. We agree that the original manuscript did not sufficiently analyze the sensitivity of the proposed hyper-parameters. Although the roles of $\alpha$, $\beta$, $k$, $\gamma$, $k_{\mathrm{root}}$, and $k_{\mathrm{tip}}$ were described in the method section, the original version only provided a brief qualitative discussion and did not systematically evaluate how these parameters affect visual quality, deformation stability, temporal coherence, and computational cost.

To address this concern, we substantially revised the **Parameter Sensitivity** subsection. Rather than simply adding a short explanation, we replaced the previous brief discussion with a systematic parameter sensitivity analysis. In the revised manuscript, we vary one parameter at a time while keeping the others fixed to the default setting. The evaluated parameters include $\alpha$, $\beta$, $k$, $\gamma$, $k_{\mathrm{root}}$, and $k_{\mathrm{tip}}$. For each setting, we report Strand Displacement Error, Deformation Error, Motion Variance, Temporal Consistency Error, and Runtime.

The added analysis clarifies the role of each parameter. The parameter $\alpha$ controls the transition width of the low-frequency sigmoid kernel. When $\alpha$ is too small, the interpolation becomes overly smooth and motion attenuation increases. When $\alpha$ is too large, the interpolation response becomes sharper and can slightly increase temporal inconsistency. The parameter $\beta$ controls the sharpness of the high-frequency tanh-based kernel. Larger $\beta$ values reduce mid-region velocity cancellation, but excessively large values may introduce abrupt changes in interpolation weights, resulting in increased deformation error or temporal jitter.

We also analyzed the exaggeration-related parameters $k$ and $k_{\mathrm{tip}}$, which have the strongest influence on visual expressiveness. Increasing these values improves strand-level motion diversity and curl separation, as reflected by higher Motion Variance and lower Strand Displacement Error. However, overly large values can also increase Deformation Error and Temporal Consistency Error due to excessive motion amplification. Therefore, the revised manuscript explains that moderate values provide the best balance between expressiveness and stability.

In addition, we evaluated the root–tip adaptation parameters $\gamma$, $k_{\mathrm{root}}$, and $k_{\mathrm{tip}}$. The results show that keeping $k_{\mathrm{root}}$ small or non-positive helps maintain root stability, while using a positive $k_{\mathrm{tip}}$ enhances expressive motion near the strand tip. The parameter $\gamma$ controls how strongly the exaggeration is concentrated toward the tip. A moderate value of $\gamma$ provides a good compromise between stable root behavior and expressive tip motion.

The added sensitivity analysis also shows that runtime is only weakly affected by parameter changes, because the number of kernel evaluations and normalization operations remains the same across parameter settings. Based on this analysis, we use a fixed default parameter setting across all experiments without per-scene tuning. This demonstrates that the proposed framework has a stable operating range and can generalize across different hair models and upsampling scenarios.

Accordingly, the revised manuscript now provides a systematic evaluation of how the proposed hyper-parameters affect simulation quality and stability. We believe this revision directly addresses the reviewer’s concern and makes the parameter behavior of the proposed method clearer and more reproducible.

**Revision:** We replaced the previous brief **Parameter Sensitivity** subsection with an expanded systematic analysis. The revised subsection includes a one-factor-at-a-time parameter sweep for $\alpha$, $\beta$, $k$, $\gamma$, $k_{\mathrm{root}}$, and $k_{\mathrm{tip}}$. We also added a new table reporting Strand Displacement Error, Deformation Error, Motion Variance, Temporal Consistency Error, and Runtime for different parameter settings, together with a discussion explaining how each parameter affects expressiveness, deformation stability, temporal coherence, and computational efficiency. (see page 24 in revised manuscript: **"Parameter Sensitivity …"**)

**Reviewer#1, Concern#6:** The computational complexity of the kernel proposed should be evaluated. Comparing the runtime, memory usage and scalability with growing strand numbers would be very helpful to the manuscript.

**Author response:** We thank the reviewer for this helpful suggestion. We agree that the original manuscript did not sufficiently analyze the computational complexity, memory usage, and scalability of the proposed kernel. Although we described the proposed framework as a lightweight guide–detail mapping extension, the previous version did not provide a detailed complexity analysis or scalability evaluation with increasing numbers of strands.

To address this concern, we added a new subsection, **Computational Complexity and Scalability**, to the revised manuscript. In this subsection, we provide both a theoretical complexity analysis and an empirical scalability evaluation. Specifically, we compare the proposed method with conventional linear interpolation in terms of runtime, memory usage, and scalability as the number of ghost strands increases.

The proposed method is applied only to the guide–detail mapping stage and does not introduce additional physical simulation, collision detection, constraint solving, or neural inference. Let $M$ be the number of ghost strands and $N+1$ be the number of particles per strand. Since each ghost-strand particle is updated independently, the computational complexity of the proposed method is $O(MN)$, which is the same asymptotic complexity as conventional linear interpolation. The difference is a small constant-factor overhead caused by the evaluation of the sigmoid/tanh kernels, root–tip adaptive weighting, and positive-only normalization.

We also added a scalability table that reports runtime and memory usage for different numbers of ghost strands. The results show that both linear interpolation and the proposed method scale approximately linearly as the number of ghost strands increases. The proposed method introduces a consistent overhead of approximately 13–14% compared with linear interpolation. For example, at 10k ghost strands, the runtime increases from 3.42 ms/frame to 3.89 ms/frame. This demonstrates that the proposed kernel improves motion expressiveness while maintaining practical computational efficiency.

In terms of memory usage, the proposed method does not require additional solver states, collision buffers, dense matrices, neural-network parameters, or training data. Only a small number of temporary scalar values are required for kernel evaluation and normalization. Therefore, the additional memory overhead remains small and grows only linearly with the number of ghost strands. In the revised manuscript, we also report the additional memory usage under increasing strand counts to clarify this behavior.

These results demonstrate that the proposed exaggerated-kernel framework preserves the scalability of conventional guide–detail interpolation while improving strand separation, curl preservation, and motion expressiveness with limited runtime and memory overhead. We believe that this additional analysis directly addresses the reviewer’s concern and strengthens the practical evaluation of the proposed method.

**Revision:** We added a new subsection, **Computational Complexity and Scalability**, to the Experimental Results/Discussion section. This subsection includes theoretical complexity analysis, runtime comparison, memory usage, and scalability evaluation with increasing numbers of ghost strands. We also added a new table reporting runtime and additional memory usage for different strand counts. (see page 29 in revised manuscript: **"Computational Complexity and Scalability …"**)

**Reviewer#1, Concern#7:** While the paper includes a discussion of stability in the physical sense, there are not many quantitative data supporting motion stability of this kind. Further experiments with fast head motion, collision cases or complex hairstyles would be more suitable to justify robustness.

**Author response:** We thank the reviewer for this important comment. We agree that the original manuscript did not provide sufficient quantitative evidence for motion stability under challenging conditions. We also agree that the term “stability” should be clarified more carefully in the context of the proposed method.

In the revised manuscript, we clarified that the proposed method should not be interpreted as a full physics-based stability model or a replacement for a hair dynamics solver. Rather, the proposed framework is a geometric and kinematic guide–detail mapping method that modifies how ghost strands interpolate the motion of simulated guide strands. Therefore, the stability evaluated in this work refers to motion stability under exaggerated interpolation, including suppression of excessive stretching, temporal jitter, frame-to-frame discontinuity, and root instability. The underlying physical validity of guide-strand simulation remains determined by the original solver, such as PBD, XPBD, or projective dynamics.

To address the reviewer’s concern, we added a new robustness evaluation under challenging motion and hairstyle scenarios. Specifically, we tested the proposed method under fast head motion, collision-like guide perturbation, complex curly hairstyles, and dense coiled hairstyles. In the fast head motion scenario, guide strands are driven by rapidly changing head orientations and high angular velocities. In the collision-like perturbation scenario, localized impulse-like disturbances are applied to guide strands to approximate abrupt contact-induced motion. Although full strand–strand collision handling is outside the scope of the proposed kernel formulation, this experiment allows us to evaluate whether the guide–detail interpolation remains stable under sudden local motion changes.

We added quantitative measurements including Deformation Error, Maximum Stretch Ratio, Temporal Consistency Error, Frame-to-Frame Stability Error, and Failure Rate. The results show that fast head motion and collision-like perturbations increase deformation and temporal errors compared with normal motion, as expected. However, the maximum stretch ratio remains bounded in all tested cases, and the failure rate remains low. This indicates that the proposed positive-only normalization, root–tip adaptive exaggeration, and hybrid update effectively suppress excessive stretching and unstable frame-to-frame behavior even under challenging guide motions.

We also evaluated complex curly and dense coiled hairstyle cases. These cases produce slightly higher deformation and temporal errors due to stronger local curvature and denser strand variation. Nevertheless, the proposed method remains stable and preserves strand separation without producing severe jitter or root clustering. These additional results support the robustness of the proposed geometric exaggerated-kernel framework under challenging guide motions and complex hairstyle configurations.

In addition, we revised the **Limitations** section to explicitly state the scope of the proposed method. The revised manuscript now clarifies that the proposed kernel does not explicitly solve collision response, frictional contact, self-contact, or fluid coupling between strands. These effects remain dependent on the underlying physical solver. Therefore, the proposed method is complementary to existing physics-based solvers and focuses on improving the stability and expressiveness of ghost-strand interpolation.

**Revision:** We added a new subsection, **Robustness under Challenging Motions**, which includes additional experiments with fast head motion, collision-like guide perturbation, complex curly hairstyles, and dense coiled hairstyles. We also added quantitative robustness metrics, including Deformation Error, Maximum Stretch Ratio, Temporal Consistency Error, Frame-to-Frame Stability Error, and Failure Rate. In addition, we revised the **Limitations** section to clarify that the proposed method is a geometric/kinematic guide–detail mapping framework rather than a full physics-based stability model.

(see page 27 in revised manuscript: **"Robustness under Challenging Motions …"**)

(see page 32 in revised manuscript: **"Limitations …"**)

**Reviewer#1, Concern#8:** The figures would be clearer if they were higher resolution or larger size font.

**Author response:** We thank the reviewer for this helpful suggestion. We agree that some figures in the original manuscript were difficult to read because the rendered hair strands, plot labels, and visual details appeared relatively small in the submitted version.

To address this concern, we revised the figures throughout the manuscript. Specifically, we replaced the major visual result figures with larger and higher-resolution versions so that strand-level details, curl structures, strand separation, and volumetric differences can be more clearly observed. We also enlarged the plot figures and increased the relative size of labels, tick marks, legends, and curve thickness where applicable, especially in the kernel analysis figures.

For the hair simulation result figures, we increased the image resolution and enlarged the rendered results to improve the visibility of fine strand structures. This makes the comparison between the baseline interpolation and the proposed method clearer, particularly in the curly, coiled, short-hair, and dense hairstyle examples. For the kernel-related figures, we increased the figure size and improved the visibility of the curves and reference lines so that the interpolation behavior and mid-region cancellation effect are easier to understand.

We did not substantially modify the captions because the meaning, comparison structure, and experimental content of the figures remain unchanged. The revision was focused on improving visual clarity and readability rather than changing the interpretation of the figures.

**Revision:** We replaced the main figures with larger and higher-resolution versions. We also enlarged the plot elements, labels, legends, and visual results where necessary to improve readability. The captions were retained because the figure content and comparisons remain the same.

**Reviewer#1, Concern#9:** There are a few interrelated works published in 2023-2026 that relate to the neural hair simulation and real-time strand interpolation, which should be referenced in order to better place the contribution.

**Author response:** We thank the reviewer for pointing this out. We agree that the manuscript should more clearly position the proposed method with respect to recent neural hair simulation and real-time strand interpolation studies. Although the previous version already discussed guide-based tracking and interpolation methods, the relationship to the most recent neural, physically guided, and real-time hair simulation approaches was not sufficiently explicit.

To address this concern, we revised the related discussion by adding a paragraph titled **Comparison with Recent Hair Simulation Methods**. In this paragraph, we discuss recent guide-based, learning-based, and physically guided hair simulation methods, including neural interpolation, physically guided hair interpolation, neural real-time hair deformation, and dense physics-based strand simulation. These references help clarify how recent studies have advanced hair simulation through learned motion reconstruction, force-aware interpolation, and extended physical models.

We also clarified the difference between these methods and our proposed framework. Recent neural approaches can generate high-quality hair deformation and detail motion, but they generally require training data and neural inference. Physically guided interpolation methods improve physical plausibility by reconstructing rendered hair motion from internal forces or material models, but they require additional force-level reconstruction or constraint-solving processes. Dense physics-based strand simulation methods improve physical stability, but they modify or extend the underlying simulation model.

In contrast, our method addresses a complementary problem: the loss of expressiveness caused by bounded guide-to-detail interpolation weights. The proposed exaggerated-kernel framework does not introduce a new physical solver or a learned network. Instead, it analytically modifies the interpolation coefficient in the guide–detail mapping stage to alleviate mid-region velocity cancellation and enhance strand separation, fluttering, and curl preservation. Therefore, our method can be used as a lightweight analytical component within modern guide-based, neural, or physics-based hair simulation pipelines.

**Revision:** We added and refined the paragraph **Comparison with Recent Hair Simulation Methods** to include recent interrelated works on neural hair simulation, physically guided real-time interpolation, and dense physics-based strand simulation. We also clarified that the proposed method is complementary to these recent approaches and focuses specifically on the interpolation-weight artifact in guide–detail tracking pipelines. (see page 9 in revised manuscript: **"Comparison with Recent Hair Simulation …"**)

**Reviewer#1, Concern#10:** The discussion section should discuss the constraint that this proposed framework has, such as when there are artifacts introduced from exaggerated interpolation.

**Author response:** We thank the reviewer for this valuable comment. We agree that the original manuscript did not sufficiently discuss the practical constraints of the proposed framework, especially the artifacts that may be introduced by exaggerated interpolation under inappropriate parameter settings or challenging guide motions.

To address this concern, we added a new discussion subsection, **Constraints and Artifacts of Exaggerated Interpolation**, to the revised manuscript. In this subsection, we explicitly discuss the conditions under which the proposed exaggerated-kernel interpolation may produce artifacts. Although the proposed method improves ghost-strand expressiveness by alleviating mid-region motion cancellation, it intentionally amplifies relative motion differences between guide strands. Therefore, when the exaggeration strength is too large or when guide motion changes abruptly, artifacts such as over-stretching, length divergence, curl distortion, and temporal jitter may occur.

We also clarified the parameter-related constraints of the method. In particular, large values of $k$ or $k_{\mathrm{tip}}$ may excessively amplify strand motion and increase deformation artifacts, while an overly sharp high-frequency transition controlled by $\beta$ may increase frame-to-frame variation. This discussion is now connected to the added parameter sensitivity analysis and robustness evaluation, where deformation error, temporal consistency, and frame-to-frame stability are quantitatively evaluated.

In addition, we discuss short-hair-specific artifacts. In short-hair configurations, root-position bias can strongly affect the final strand distribution, and direct exaggerated interpolation of absolute strand positions may cause root clustering. To clarify how this issue is handled, we added an explanation that the proposed root-decoupled interpolation strategy separates exaggerated strand shape from final root placement, thereby reducing root clustering artifacts.

We also discuss the limitation related to guide–detail correspondence. The proposed method assumes reasonably smooth and stable correspondence between guide strands and ghost strands. If guide strands are highly irregular, topologically inconsistent, or affected by severe collision and self-contact, the exaggerated kernel may amplify correspondence errors. Therefore, we clarified that the proposed framework should be regarded as a lightweight guide–detail mapping method for improving interpolation expressiveness, rather than a complete solution for all physical interaction artifacts.

Finally, we explained how the proposed stabilization components mitigate these artifacts. Positive-only normalized weights suppress negative-weight amplification and excessive stretching. Root–tip adaptive exaggeration limits unstable motion near the root while preserving expressive motion near the tip. The hybrid update further reduces length divergence by blending velocity-based motion with position-based stabilization.

**Revision:** We added a new subsection, **Constraints and Artifacts of Exaggerated Interpolation**, to the Discussion section. This subsection discusses possible artifacts caused by exaggerated interpolation, including over-stretching, length divergence, curl distortion, temporal jitter, root clustering, and correspondence-related errors. We also explain how positive-only normalization, root–tip adaptive exaggeration, hybrid update, and root-decoupled interpolation mitigate these artifacts. (see page 32 in revised manuscript: **"Constraints and Artifacts of …"**)

**Reviewer#1, Concern#11:** There is a very helpful notation table, but some symbols are used before they are properly defined. Consider ensuring notation is introduced consistently.

**Author response:** We thank the reviewer for this helpful comment. We agree that, although the manuscript includes a notation table, some symbols in the original version were used before being explicitly introduced in the main text. This could make the mathematical formulation harder to follow, especially in the method and quantitative evaluation sections.

To address this concern, we carefully reviewed the notation throughout the manuscript and revised the order in which symbols are introduced. In the revised version, the notation table is retained as a summary reference, but the main text now also provides local definitions when important symbols first appear. This allows readers to understand the formulation sequentially without relying only on the notation table.

Specifically, we revised the **Notation and Setup** subsection to explicitly define the guide strands $A$ and $B$, the ghost strand $G$, the particle index $i$, the number of strand particles $N+1$, and the position and velocity variables $\mathbf{x}$ and $\mathbf{v}$ before they are used in the interpolation equations. We also clarified the definition of the root-based mixing coordinate $s$ and the symmetric kernel input coordinate $u$ before introducing the kernel functions.

In addition, we revised the quantitative evaluation and robustness sections to ensure that all metric symbols are introduced before being reported in equations or tables. In particular, we now define $E_{\mathrm{disp}}$, $E_{\mathrm{traj}}$, $E_{\mathrm{def}}$, $V_{\mathrm{motion}}$, $E_{\mathrm{temp}}$, and $E_{\mathrm{stab}}$ at the beginning of the quantitative evaluation section. We also explicitly define $R_{\mathrm{stretch}}$ before presenting the maximum stretch ratio equation in the robustness evaluation.

We believe these revisions improve the consistency and readability of the notation and make the mathematical formulation easier to follow.

**Revision:** We revised the notation usage throughout the manuscript so that all major symbols are introduced before they are used. We updated the **Notation and Setup** subsection and added local definitions for the main variables, kernel coordinates, evaluation metrics, and robustness metrics. The notation table remains as a compact reference, while the text now introduces symbols more consistently.

(see page 10 in revised manuscript: **"Notation and Setup …"**)

(see page 10 in revised manuscript: **"Before introducing the …"**)

(see page 10 in revised manuscript: **"We use** $t(u)$ **to …"**)

(see page 11 in revised manuscript: **"For root-tip adaptation, we …"**)

(see page 18 in revised manuscript: **"For clarity, all …"**)

**Reviewer#1, Concern#12:** The final section should provide more explicit suggestions for future work, such as incorporating with neural simulation packages, GPU implementation and animation pipeline production.

**Author response:** We thank the reviewer for this constructive suggestion. We agree that the final section of the original manuscript did not provide sufficiently explicit future work directions. In the previous version, future work was mentioned only briefly, mainly in terms of adaptive parameter selection and possible integration with learning-based approaches.

To address this concern, we revised the **Conclusion** section and expanded the future work discussion with more concrete directions. Specifically, we now discuss adaptive parameter selection, integration with neural hair simulation and neural interpolation systems, GPU implementation, and incorporation into practical animation and production pipelines.

First, we added a future direction on adaptive parameter selection. Since the proposed framework includes several controllable parameters, such as $\alpha$, $\beta$, $k$, $\gamma$, $k_{\mathrm{root}}$, and $k_{\mathrm{tip}}$, we plan to investigate automatic parameter selection based on local strand geometry, guide-strand motion, and hairstyle characteristics. This would reduce empirical tuning and make the method easier to apply across diverse hair models.

Second, we explicitly discuss integration with neural hair simulation and neural interpolation systems. Because the proposed method operates only at the guide–detail mapping stage, it can be combined with learned guide prediction, neural strand interpolation, or data-driven hair deformation frameworks as an analytical post-processing or control layer. This direction could combine the controllability of the proposed exaggerated kernel with the high-quality detail reconstruction capability of neural methods.

Third, we added GPU implementation as a concrete future direction. The proposed kernel evaluation, root–tip weighting, positive-only normalization, and ghost-strand update are all local per-strand or per-particle operations. Therefore, the framework is well suited for massively parallel GPU execution. A GPU implementation would further reduce runtime overhead and enable real-time use in interactive applications, virtual humans, games, and XR environments.

Finally, we expanded the future work discussion to include practical animation and production pipeline integration. In production environments, guide hairs are commonly simulated first and dense render hairs are generated through interpolation or tracking. Since the proposed framework does not require modifying the underlying physical solver, it can be incorporated as a lightweight guide–detail mapping module for improving strand separation, curl preservation, and stylized motion expressiveness. This makes the method suitable for artist-controllable hair animation, real-time character systems, and production-oriented grooming workflows.

We believe that these revisions make the final section more concrete and better clarify how the proposed framework can be extended beyond the current implementation.

**Revision:** We revised the **Conclusion** section to provide more explicit future work directions, including adaptive parameter selection, integration with neural hair simulation systems, GPU implementation, and incorporation into animation and production pipelines. (see page 33 in revised manuscript: **"As future work, we …"**)

**Reviewer#2, Concern#1:** For the parameter sensitivity, it seems that only qualitative descriptions are provided. It will be good if the authors can provide more quantitative results to justify their claims about the sensitivity.

**Author response:** We thank the reviewer for this helpful comment. We agree that parameter sensitivity should be supported by quantitative evidence rather than only qualitative descriptions.

In the revised manuscript, we have already expanded the **Parameter Sensitivity** subsection to include a quantitative parameter sweep for the main hyper-parameters of the proposed framework, including $\alpha$, $\beta$, $k$, $\gamma$, $k_{\mathrm{root}}$, and $k_{\mathrm{tip}}$. We used a one-factor-at-a-time evaluation strategy, where one parameter was varied while the remaining parameters were fixed to the default setting. This allows us to isolate the effect of each parameter on the quality and stability of the simulation.

For each parameter setting, we reported multiple quantitative metrics: Strand Displacement Error, Deformation Error, Motion Variance, Temporal Consistency Error, and Runtime. These metrics were selected to evaluate different aspects of the method, including motion preservation, deformation stability, strand-level motion diversity, temporal coherence, and computational cost.

The added numerical results show clear trends. For example, increasing the exaggeration strength $k$ improves motion diversity and strand separation, as reflected by higher Motion Variance. However, overly large values of $k$ also increase Deformation Error and Temporal Consistency Error, indicating that excessive exaggeration can lead to stretching artifacts or temporal jitter. A similar tendency is observed for $k_{\mathrm{tip}}$, where stronger tip exaggeration improves expressive motion near the strand tips but can increase deformation if the value is too large.

The results also clarify the roles of $\alpha$ and $\beta$. Smaller values lead to smoother but more averaged interpolation, while larger values increase the sharpness of the kernel transition. However, overly sharp transitions can increase temporal inconsistency. In addition, the analysis of $\gamma$ shows how the root–tip adaptation profile affects the balance between root stability and expressive tip motion.

Overall, the quantitative sensitivity analysis supports our claim that the proposed framework has a stable operating range. It also shows that the selected default parameters provide a practical balance between visual expressiveness, deformation stability, temporal coherence, and computational efficiency.

**Revision:** No additional manuscript changes were made for this concern beyond the already revised **Parameter Sensitivity** subsection. The revised manuscript already includes a quantitative parameter sensitivity table reporting Strand Displacement Error, Deformation Error, Motion Variance, Temporal Consistency Error, and Runtime for different values of $\alpha$, $\beta$, $k$, $\gamma$, $k_{\mathrm{root}}$, and $k_{\mathrm{tip}}$. (see page 24 in revised manuscript: **"Parameter Sensitivity …"**)

**Reviewer#2, Concern#2:** Following the PLOS Data policy, the data should be "provided as part of the manuscript or its supporting information, or deposited to a public repository". The authors should therefore provide at least a reviewer-accessible repository for the review process and a public repository later.

**Author response:** We thank the reviewer for pointing out this important issue regarding the PLOS Data policy. We agree that the data and code supporting the findings of the manuscript should be accessible during the review process, rather than being made available only after acceptance.

To address this concern, we revised the **Data and Code Availability** section of the manuscript. The source code, experimental scripts, parameter settings, and representative hair model data used in this study are now publicly available through the following GitHub repository:

https://github.com/dohee02/HairSimulation-ExaggerationKernel.git

The repository is available during the review process and will remain publicly accessible after publication. Therefore, reviewers can access the materials needed to verify the implementation and reproduce the main results reported in the manuscript.

The repository includes the implementation of the proposed nonlinear exaggerated kernel framework, including the baseline sigmoid interpolation, high-frequency transition kernel, exaggerated kernel construction, positive-only normalized stabilization, root–tip adaptive exaggeration, and hybrid update strategy. It also includes parameter settings, representative input hair models, experimental scripts, and documentation describing the expected input and output formats.

In addition, the released materials include scripts and data required to reproduce the qualitative comparisons, quantitative evaluation, statistical analysis, robustness evaluation, and visual comparison results reported in the manuscript. These materials allow reviewers and readers to verify the effects of the proposed kernel design on curl preservation, strand separation, volumetric richness, suppression of mid-region motion cancellation, temporal stability, and computational overhead.

Accordingly, the revised manuscript now complies with the PLOS Data policy by providing the supporting code and data through a public repository that is accessible during review and will remain available after publication.

**Revision:** No additional manuscript changes were made for this concern beyond the already revised **Data and Code Availability** section. The revised manuscript now provides a public GitHub repository containing the source code, experimental scripts, parameter settings, representative hair model data, and reproduction materials. (see page 34 in revised manuscript: **"Data and Code Availability …"**)

**Reviewer#2, Concern#3:** Page 3, Figure 2: The indistinguishable orange lines may lead to some confusion. It will be better to use dashed lines or similar line types that can show two sets of curves at the same position.

**Author response:** We thank the reviewer for this helpful suggestion. We agree that the overlapping curves in the original Figure 2 could cause confusion. In particular, the exaggerated kernel and the stabilized kernel were very close to each other in several regions, and the original figure did not make this overlap sufficiently clear.

To address this concern, we revised Figure 2 by replacing it with a larger and higher-resolution version. The revised figure improves the visibility of the curves, labels, and plot details, making the relationship between the exaggerated and stabilized kernels easier to understand. The enlarged and higher-resolution plot also makes it clearer that the stabilized kernel closely follows the exaggerated kernel while enforcing stable interpolation behavior.

Since this issue was related to figure readability rather than the method or experimental interpretation, we did not add new explanatory text to the manuscript body. Instead, we improved the figure itself so that the curve behavior can be more clearly observed.

**Revision:** We replaced Figure 2 with a larger and higher-resolution version to improve the visibility of overlapping curves and reduce possible confusion.

**Reviewer#2, Concern#4:** Page 5, Line 133: The section information is missing in “The detailed contributions are summarized in Section .”

**Author response:** We thank the reviewer for pointing out this formatting and cross-reference issue. We agree that the incomplete section reference could confuse readers and should be corrected.

In the revised manuscript, we checked the entire document for similar incomplete or missing cross-references and corrected them consistently. In particular, the sentence “The detailed contributions are summarized in Section .” has been revised to avoid the missing reference. Since the contribution summary appears immediately after that paragraph, we changed the sentence to a direct transition: “The main contributions are summarized below.”

We also reviewed other section-reference expressions in the manuscript and removed or corrected incomplete references where necessary. This ensures that no unresolved “Section .” style references remain in the revised version.

**Revision:** We corrected the incomplete section reference on Page 5, Line 133, and checked the manuscript for similar cross-reference errors. The revised manuscript now uses complete and clear transition sentences instead of unresolved section references.

**Reviewer#3, Concern#1:** Reviewer #3: The authors propose a nonlinear exaggerated kernel framework that replaces the conventional [0,1]-bounded interpolation weight used in guide-strand-based hair upsampling. By linearly combining a low-frequency sigmoid kernel t_L(u) and a high-frequency tanh kernel t_H(u) with an enhancement factor k > 0, the resulting kernel t_ex(u) = (1+k) t_H(u) − k t_L(u) can exceed [0,1] in the mid-region. This is intended to alleviate the velocity-cancellation artifact (t ≈ 0.5) that occurs when two neighboring guide strands move in opposite directions. The authors further introduce (1) a positive-only normalized stabilization step to prevent over-stretch when weights become negative, (2) a root–tip adaptive scheme that scales k along the strand, and (3) a root-decoupled hybrid update for short-hair configurations where root clustering would otherwise occur. The method is positioned as a solver-agnostic extension layer compatible with PBD/XPBD/projective-dynamics solvers. Strengths, Clear problem formulation, Mathematically clean construction, Solver agnostic, Stabilization is well motivated, Qualitative results are visually convincing across curly, coiled, short, and diverse hair models.

**Author response:** We sincerely thank the reviewer for the positive and encouraging evaluation of our work. We appreciate the reviewer’s recognition of the clear problem formulation, mathematically clean construction, solver-agnostic design, stabilization strategy, and visually convincing qualitative results across diverse hair models. Since no specific concerns were raised in this review, we have carefully considered the reviewer’s summary and strengths while revising the manuscript to further improve clarity, quantitative validation, and reproducibility in response to the other reviewers’ comments.
