# Supplementary material for: A nonlinear exaggerated kernel framework for expressive hair motion in tracking solution-based simulation
Source: PLoS One. 2026 Aug 31;21(8):e0356631. doi: 10.1371/journal.pone.0356631 (PMC13529024; doi:10.1371/journal.pone.0356631)
Supplement: Peer review history

# Peer review: Decision Letter 0

**DOI:** 10.1371/journal.pone.0356631.r001

**Authors:** Marco Antonio Moreno-Armendariz

29 Jun 2026

Dear Dr. Kim,

Thank you for submitting your manuscript to PLOS ONE. After careful consideration, we feel that it has merit but does not fully meet PLOS ONE’s publication criteria as it currently stands. Therefore, we invite you to submit a revised version of the manuscript that addresses the points raised during the review process.

Please submit your revised manuscript by Aug 28 2026 11:59PM. If you will need more time than this to complete your revisions, please reply to this message or contact the journal office at plosone@plos.org. When you're ready to submit your revision, log on to https://www.editorialmanager.com/pone/ and select the 'Submissions Needing Revision' folder to locate your manuscript file.

- A letter that responds to each point raised by the academic editor and reviewer(s). You should upload this letter as a separate file labeled 'Response to Reviewers'.
- A marked-up copy of your manuscript that highlights changes made to the original version. You should upload this as a separate file labeled 'Revised Manuscript with Track Changes'.
- An unmarked version of your revised paper without tracked changes. You should upload this as a separate file labeled 'Manuscript'.

If you would like to make changes to your financial disclosure, please include your updated statement in your cover letter. Guidelines for resubmitting your figure files are available below the reviewer comments at the end of this letter.

If applicable, we recommend that you deposit your laboratory protocols in protocols.io to enhance the reproducibility of your results. Protocols.io assigns your protocol its own identifier (DOI) so that it can be cited independently in the future. For instructions see: https://journals.plos.org/plosone/s/submission-guidelines#loc-laboratory-protocols. Additionally, PLOS ONE offers an option for publishing peer-reviewed Lab Protocol articles, which describe protocols hosted on protocols.io. Read more information on sharing protocols at https://plos.org/protocols?utm_medium=editorial-email&utm_source=authorletters&utm_campaign=protocols.

As the corresponding author, your ORCID iD is verified in the submission system and will appear in the published article. PLOS supports the use of ORCID, and we encourage all coauthors to register for an ORCID iD and use it as well. Please encourage your coauthors to verify their ORCID iD within the submission system before final acceptance, as unverified ORCID iDs will not appear in the published article. *Only*  the individual author can complete the verification step; PLOS staff *cannot*  verify ORCID iDs on behalf of authors.

We look forward to receiving your revised manuscript.

Kind regards,

Marco Antonio Moreno-Armendariz, Ph.D.

Academic Editor

PLOS One

Journal Requirements:

When submitting your revision, we need you to address these additional requirements.

1. Please ensure that your manuscript meets PLOS ONE's style requirements, including those for file naming. The PLOS ONE style templates can be found at

https://journals.plos.org/plosone/s/file?id=wjVg/PLOSOne_formatting_sample_main_body.pdf and https://journals.plos.org/plosone/s/file?id=ba62/PLOSOne_formatting_sample_title_authors_affiliations.pdf

2. Please note that PLOS One has specific guidelines on code sharing for submissions in which author-generated code underpins the findings in the manuscript. In these cases, we expect all author-generated code to be made available without restrictions upon publication of the work. Please review our guidelines at https://journals.plos.org/plosone/s/materials-and-software-sharing#loc-sharing-code and ensure that your code is shared in a way that follows best practice and facilitates reproducibility and reuse.

3. In the online submission form, you indicated that your data will be submitted to a repository upon acceptance. We strongly recommend all authors deposit their data before acceptance, as the process can be lengthy and hold up publication timelines. Please note that, though access restrictions are acceptable now, your entire minimal dataset will need to be made freely accessible if your manuscript is accepted for publication. This policy applies to all data except where public deposition would breach compliance with the protocol approved by your research ethics board. If you are unable to adhere to our open data policy, please kindly revise your statement to explain your reasoning and we will seek the editor's input on an exemption.

4. Thank you for stating the following financial disclosure:

This work was supported by INHA UNIVERSITY Research Grant.

This work was supported by the National Research Foundation of Korea(NRF) grant funded by the Korea government(MSIT) (RS-2026-25474535).

Please state what role the funders took in the study. If the funders had no role, please state: "The funders had no role in study design, data collection and analysis, decision to publish, or preparation of the manuscript."

If this statement is not correct you must amend it as needed.

Please include this amended Role of Funder statement in your cover letter; we will change the online submission form on your behalf.

5. We note that Figure(s) 4, 5, 6, 7, 8, 9, 10, 11 in your submission contain copyrighted images. All PLOS content is published under the Creative Commons Attribution License (CC BY 4.0), which means that the manuscript, images, and Supporting Information files will be freely available online, and any third party is permitted to access, download, copy, distribute, and use these materials in any way, even commercially, with proper attribution. For more information, see our copyright guidelines: http://journals.plos.org/plosone/s/licenses-and-copyright.

We require you to either (1) present written permission from the copyright holder to publish these figures specifically under the CC BY 4.0 license, or (2) remove the figures from your submission:

a. You may seek permission from the original copyright holder of Figure(s) 4, 5, 6, 7, 8, 9, 10, 11  to publish the content specifically under the CC BY 4.0 license.

We recommend that you contact the original copyright holder with the Content Permission Form (http://journals.plos.org/plosone/s/file?id=7c09/content-permission-form.pdf) and the following text:

“I request permission for the open-access journal PLOS ONE to publish XXX under the Creative Commons Attribution License (CCAL) CC BY 4.0 (http://creativecommons.org/licenses/by/4.0/). Please be aware that this license allows unrestricted use and distribution, even commercially, by third parties. Please reply and provide explicit written permission to publish XXX under a CC BY license and complete the attached form.”

Please upload the completed Content Permission Form or other proof of granted permissions as an "Other" file with your submission.

In the figure caption of the copyrighted figure, please include the following text: “Reprinted from [ref] under a CC BY license, with permission from [name of publisher], original copyright [original copyright year].”

b. If you are unable to obtain permission from the original copyright holder to publish these figures under the CC BY 4.0 license or if the copyright holder’s requirements are incompatible with the CC BY 4.0 license, please either i) remove the figure or ii) supply a replacement figure that complies with the CC BY 4.0 license. Please check copyright information on all replacement figures and update the figure caption with source information. If applicable, please specify in the figure caption text when a figure is similar but not identical to the original image and is therefore for illustrative purposes only.

6. Please upload a new copy of Figure 1, 2, 3,  as the detail is not clear. Please follow the link for more information: https://journals.plos.org/plosone/s/figures

7. If the reviewer comments include a recommendation to cite specific previously published works, please review and evaluate these publications to determine whether they are relevant and should be cited. There is no requirement to cite these works unless the editor has indicated otherwise.

Additional Editor Comments :

Dear Author,

Please fill in the reviewer's comments. In particular, the sensitivity study.

Best wishes,

Marco.

[Note: HTML markup is below. Please do not edit.]

Reviewers' comments:

Reviewer's Responses to Questions

**Comments to the Author**

1. Is the manuscript technically sound, and do the data support the conclusions?

Reviewer #1: Partly

Reviewer #2: Yes

Reviewer #3: Partly

**********

2. Has the statistical analysis been performed appropriately and rigorously?

Reviewer #1: No

Reviewer #2: Yes

Reviewer #3: No

**********

3. Have the authors made all data underlying the findings in their manuscript fully available?

The PLOS Data policy

Reviewer #1: No

Reviewer #2: No

Reviewer #3: No

**********

4. Is the manuscript presented in an intelligible fashion and written in standard English?

Reviewer #1: Yes

Reviewer #2: Yes

Reviewer #3: Yes

**********

Reviewer #1: This manuscript proposes a nonlinear exaggerated kernel framework to enhance ghost strand interpolation in tracking solution-based hair simulation. The proposed method presents a novel idea, combining high-frequency enhancement and kernel interpolation to reduce the effect of momentum cancellation in the mid-frequency region and achieve numerical stability with positive kernel normalization and root-tip adaptive weighting. The manuscript is well-motivated, technically comprehensible and are well organized.

Prior to publication, there are several important points that need to be discussed.

Major Comments

1.The experimental validation is mainly qualitative. While the comparisons in the visual sense show increased expressiveness, there is a lack of quantitative evaluation in the manuscript. Other numerical measurements like: 2.Strand Displacement Error, Trajectory Error, Deformation Error, Motion Variance, Temporal Consistency, Frame-to-Frame Stability, Computational Overhead and Runtime analysis should be reported.

3.No statistics have been provided. More than one experimental scenario will be evaluated, so both appropriate statistics (mean, standard deviation, confidence intervals, significance tests) and effect sizes (where applicable) should be provided to demonstrate the robustness of the proposed approach.

4.Limited comparison with current and modern techniques. The manuscript should provide comparisons to recent (past 5 years) hair simulation methods based on guides, learning and physics. Comparisons of performance should include both visual quality and computational efficiency.

5.The reproducibility of the work should be improved. The manuscript indicates that source code and datasets will be made available following acceptance, but PLOS ONE demands that the underlying data which supports the findings must be made available during the review process or in a public repository. A repository link and/or a reviewer access should be offered.

6.The proposed method has several hyper-parameters (α, β, k, γ, kroot, ktip) but sensitivity analysis is not provided. A systematic evaluation of these parameters on the quality and stability of the simulations should be done.

7.The computational complexity of the kernel proposed should be evaluated. Comparing the runtime, memory usage and scalability with growing strand numbers would be very helpful to the manuscript.

While the paper includes a discussion of stability in the physical sense, there are not many quantitative data supporting motion stability of this kind. Further experiments with fast head motion, collision cases or complex hairstyles would be more suitable to justify robustness.

Minor Comments

1.The figures would be clearer if they were higher resolution or larger size font.

2.There are a few interrelated works published in 2023-2026 that relate to the neural hair simulation and real-time strand interpolation, which should be referenced in order to better place the contribution.

3.The discussion section should discuss the constraint that this proposed framework has, such as when there are artifacts introduced from exaggerated interpolation.

4.There is a very helpful notation table, but some symbols are used before they are properly defined. Consider ensuring notation is introduced consistently.

5.The final section should provide more explicit suggestions for future work, such as incorporating with neural simulation packages, GPU implementation and animation pipeline production.

Overall Recommendation

The manuscript is a promising and technically interesting contribution that can guide a guide-detail hair simulation via a novel nonlinear kernel formulation. But further quantitative validation, better experimental comparisons, statistics, reproducibility, and more extensive benchmarking are required prior to publication. So, I suggest that it be considered for a Major Revision.

Reviewer #2: This work tackles the problem of hair simulation. Conventional approaches approximate ghost strands using interpolation, which can lead to cancellations or suppressions. To resolve this issue, this work presents a new method utilizing an exaggerated kernel. In particular, the exaggerated kernel is formed by an interpolation between a high-frequency transition kernel and a low-frequency transition kernel, with the interpolation coefficients allowed to exceed 1 for amplifying the motion of ghost strands. Detailed descriptions of the setup of the exaggeration coefficient and stabilization are provided. Experimental results on curly hair, coiled hair, and short hair are presented to demonstrate the effectiveness of the method in handling different scenarios. Ablation study is also presented.

Overall, this work is interesting and technically sound, and the experimental results are convincing. I think the paper is well-suited for PLOS One.

I have some minor comments:

1.For the parameter sensitivity, it seems that only qualitative descriptions are provided. It will be good if the authors can provide more quantitative results to justify their claims about the sensitivity.

2.Following the PLOS Data policy, the data should be "provided as part of the manuscript or its supporting information, or deposited to a public repository". The authors should therefore provide at least a reviewer-accessible repository for the review process and a public repository later.

3.Page 3, Figure 2: The indistinguishable orange lines may lead to some confusion. It will be better to use dashed lines or similar line types that can show two sets of curves at the same position.

4.Page 5, Line 133: The section information is missing in “The detailed contributions are summarized in Section .”

Reviewer #3: The authors propose a nonlinear exaggerated kernel framework that replaces the conventional [0,1]-bounded interpolation weight used in guide-strand-based hair upsampling. By linearly combining a low-frequency sigmoid kernel t_L(u) and a high-frequency tanh kernel t_H(u) with an enhancement factor k > 0, the resulting kernel t_ex(u) = (1+k) t_H(u) − k t_L(u) can exceed [0,1] in the mid-region. This is intended to alleviate the velocity-cancellation artifact (t ≈ 0.5) that occurs when two neighboring guide strands move in opposite directions. The authors further introduce (i) a positive-only normalized stabilization step to prevent over-stretch when weights become negative, (ii) a root–tip adaptive scheme that scales k along the strand, and (iii) a root-decoupled hybrid update for short-hair configurations where root clustering would otherwise occur. The method is positioned as a solver-agnostic extension layer compatible with PBD/XPBD/projective-dynamics solvers.

Strengths

Clear problem formulation

Mathematically clean construction

Solver agnostic

Stabilization is well motivated

Qualitative results are visually convincing across curly, coiled, short, and diverse hair models

**********

what does this mean?). If published, this will include your full peer review and any attached files.

If you choose “no”, your identity will remain anonymous but your review may still be made public.

**Do you want your identity to be public for this peer review?**  For information about this choice, including consent withdrawal, please see our Privacy Policy

Reviewer #1: No

Reviewer #2: No

Reviewer #3: **Yes:** Satish Bhambri

**********

[NOTE: If reviewer comments were submitted as an attachment file, they will be attached to this email and accessible via the submission site. Please log into your account, locate the manuscript record, and check for the action link "View Attachments". If this link does not appear, there are no attachment files.]

To ensure your figures meet our technical requirements, please review our figure guidelines: https://journals.plos.org/plosone/s/figures

You may also use PLOS’s free figure tool, NAAS, to help you prepare publication quality figures: https://journals.plos.org/plosone/s/figures#loc-tools-for-figure-preparation.

NAAS will assess whether your figures meet our technical requirements by comparing each figure against our figure specifications.

# Author comment: Author response to Decision Letter 1

**DOI:** 10.1371/journal.pone.0356631.r002

7 Jul 2026

The “Respond to Reviewers” file has been attached in the Attach Files section.

Attachment Submitted filename: Response-to-Reviewers.docx

# Peer review: Decision Letter 1

**DOI:** 10.1371/journal.pone.0356631.r003

**Authors:** Daniel Parkes

5 Aug 2026

A Nonlinear Exaggerated Kernel Framework for Expressive Hair Motion in Tracking Solution-Based Simulation

PONE-D-26-23963R1

Dear Dr. Kim,

We’re pleased to inform you that your manuscript has been judged scientifically suitable for publication and will be formally accepted for publication once it meets all outstanding technical requirements.

Within one week, you’ll receive an e-mail detailing the required amendments. When these have been addressed, you’ll receive a formal acceptance letter and your manuscript will be scheduled for publication.

An invoice will be generated when your article is formally accepted. Please note, if your institution has a publishing partnership with PLOS and your article meets the relevant criteria, all or part of your publication costs will be covered. Please make sure your user information is up-to-date by logging into Editorial Manager at Editorial Manager® and clicking the ‘Update My Information' link at the top of the page. For questions related to billing, please contact billing support.

If your institution or institutions have a press office, please notify them about your upcoming paper to help maximize its impact. If they’ll be preparing press materials, please inform our press team as soon as possible -- no later than 48 hours after receiving the formal acceptance. Your manuscript will remain under strict press embargo until 2 pm Eastern Time on the date of publication. For more information, please contact onepress@plos.org.

Kind regards,

Daniel Parkes, PhD

Staff Editor

PLOS One

Additional Editor Comments (optional):

Reviewers' comments:

Reviewer's Responses to Questions

**Comments to the Author**

Reviewer #1: All comments have been addressed

Reviewer #2: All comments have been addressed

**********

2. Is the manuscript technically sound, and do the data support the conclusions?

Reviewer #1: Yes

Reviewer #2: Yes

**********

3. Has the statistical analysis been performed appropriately and rigorously?

Reviewer #1: Yes

Reviewer #2: Yes

**********

4. Have the authors made all data underlying the findings in their manuscript fully available?

The PLOS Data policy

Reviewer #1: Yes

Reviewer #2: Yes

**********

5. Is the manuscript presented in an intelligible fashion and written in standard English?

Reviewer #1: Yes

Reviewer #2: Yes

**********

Reviewer #1: The authors have adequately addressed the comments made at the last round of review. The revised manuscript offers a much better organized and enhanced description of the proposed nonlinear exaggerated kernel framework for a tracking solution based on hair simulation. The manuscript now contains a more compelling motivation for the proposed approach, a fuller description of related work, better explanations and notation of the methods, the details of the algorithms, and better experimental validation. The supporting quantitative assessment, statistical analysis, robustness study, discussion of computational complexity and comparison of the approach with existing work further enhances the technical quality of the work. Having the source code and supporting materials available via a public repository also enhances the reproducibility and transparency of the research. The conclusions are justified by the results presented and the manuscript is in clear, understandable English. I have no further comments on the technical parts of the paper, and it generally meets the concerns I had raised in the previous review.

I am recommending that the manuscript be published.

Reviewer #2: The authors have addressed all my comments in the revised manuscript. I am happy to recommend acceptance of the manuscript for publication.

**********

what does this mean?). If published, this will include your full peer review and any attached files.

If you choose “no”, your identity will remain anonymous but your review may still be made public.

**Do you want your identity to be public for this peer review?**  For information about this choice, including consent withdrawal, please see our Privacy Policy

Reviewer #1: No

Reviewer #2: No

**********

# Editor report: Acceptance letter

**DOI:** 10.1371/journal.pone.0356631.r004

**Authors:** Daniel Parkes

PONE-D-26-23963R1

PLOS One

Dear Dr. Kim,

I'm pleased to inform you that your manuscript has been deemed suitable for publication in PLOS One. Congratulations! Your manuscript is now being handed over to our production team.

At this stage, our production department will prepare your paper for publication. This includes ensuring the following:

* All references, tables, and figures are properly cited

* All relevant supporting information is included in the manuscript submission,

* There are no issues that prevent the paper from being properly typeset

You will receive further instructions from the production team, including instructions on how to review your proof when it is ready. Please keep in mind that we are working through a large volume of accepted articles, so please give us a few days to review your paper and let you know the next and final steps.

Lastly, if your institution or institutions have a press office, please let them know about your upcoming paper now to help maximize its impact. If they'll be preparing press materials, please inform our press team within the next 48 hours. Your manuscript will remain under strict press embargo until 2 pm Eastern Time on the date of publication. For more information, please contact onepress@plos.org.

You will receive an invoice from PLOS for your publication fee after your manuscript has reached the completed accept phase. If you receive an email requesting payment before acceptance or for any other service, this may be a phishing scheme. Learn how to identify phishing emails and protect your accounts at https://explore.plos.org/phishing.

If we can help with anything else, please email us at customercare@plos.org.

Thank you for submitting your work to PLOS One and supporting open access.

Kind regards,

PLOS ONE Editorial Office Staff

on behalf of

Dr Daniel Parkes

Staff Editor

PLOS One
